# Correlating Synthesis Conditions with Structural Evolution and Near-Infrared Emission in Glutathione-Capped Gold Nanoclusters

**DOI:** 10.1021/acsomega.6c03255

**Published:** 2026-10-12

**Authors:** Bowen Wang, Nicolas Laurand, Barbara Jachimska, Sanjay Sandhu, Daniel Doveiko, Amrutha Sankar, Christopher Kelly, Aruna Ivaturi, Yu Chen

**Affiliations:** † Photophysics Group, Centre for Molecular Nanometrology, Department of Physics, 3527 Scottish Universities Physics Alliance, University of Strathclyde, 107 Rottenrow, Glasgow G4 0NG, U.K.; ‡ Institute of Photonics, Department of Physics, SUPA, University of Strathclyde, Glasgow G1 1RD, U.K.; § Jerzy Haber Institute of Catalysis and Surface Chemistry, 132074 Polish Academy of Sciences, Krakow 30-239, Poland; ∥ Smart Material Research and Device Technology (SMaRDT) Group, Department of Pure and Applied Chemistry, University of Strathclyde, Thomas Graham Building, 293 Cathedral Street, Glasgow G1 1XL, U.K.; ⊥ School of Chemistry, 3526 University of Glasgow, University Avenue, Glasgow G12 8QQ, U.K.

## Abstract

Fluorescence imaging in the near-infrared (NIR) region has attracted increasing attention in biomedical research owing to its deep tissue penetration, reduced autofluorescence, and improved imaging contrast compared with visible-light imaging. Among NIR fluorescent probes, gold nanoclusters (AuNCs) are particularly attractive because of their size-dependent emission, excellent photostability, biocompatibility, and versatile surface chemistry. Here, we systematically investigate how reaction pH, HAuCl_4_ concentration, and temperature influence the optical properties and structural evolution of glutathione-capped gold nanoclusters (GSH-AuNCs). The combined modulation of these parameters enables fine control over the emission characteristics, driving a transition from visible to NIR fluorescence. Near-neutral pH, elevated HAuCl_4_ concentration, and higher reaction temperatures promoted the formation of NIR-emissive clusters. Under optimal conditions (95 °C, *C*_(Au)_ = 26 mM, and 80 μL of 1 M NaOH), GSH-AuNCs exhibited a single emission peak centered at 825 nm under 365 nm excitation, with a fluorescence intensity approximately 7.2-fold higher than that of conventional 640 nm-emissive GSH-AuNCs after absorption correction. Time-resolved fluorescence measurements revealed differences in excited-state dynamics among the differently emissive GSH-AuNCs, while circular dichroism spectroscopy showed systematic changes in ligand–metal coordination and Au­(I)-thiolate shell organization accompanying the emission red shift. Zeta potential and density measurements indicated precursor concentration-dependent changes in surface charge and structural compactness, suggesting that metal core growth and ligand-shell reorganization evolve in a partially decoupled manner. X-ray photoelectron spectroscopy confirmed similar elemental compositions and gold oxidation states across the differently emissive GSH-AuNCs, whereas increased Au/S ratios together with larger particle sizes determined by small-angle X-ray scattering evidenced progressive gold core growth with increasing precursor concentration. These complementary results show that the transition from red to NIR emission arises from the coupled evolution of gold core nuclearity, ligand-shell organization, and metal–ligand electronic interactions. To demonstrate biomedical applicability, GSH-AuNCs were functionalized with the SYL3C aptamer for selective detection of EpCAM-expressing PC3 cancer cells. Flow cytometry confirmed selective cellular recognition, highlighting the potential of these NIR nanoprobes for targeted cancer imaging. Overall, these findings provide a framework for engineering GSH-AuNCs with tailored structural and photophysical properties for biomedical imaging, biosensing, and image-guided therapeutic applications.

## Graphical abstract

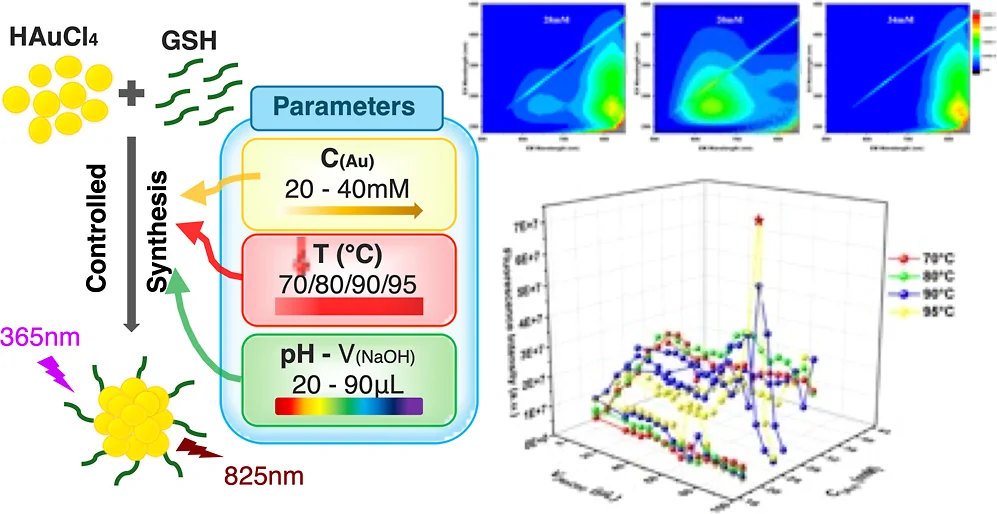

## 1 Introduction

Gold nanoclusters (AuNCs), composed of several tens of gold atoms protected by organic ligands and typically less than 2 nm in diameter, have attracted considerable attention in both fundamental research and emerging technological applications.^1–4^ Their distinctive fluorescence characteristics, including tunable emission, high photostability, biocompatibility, and long fluorescence lifetime, make them particularly promising for biomedical imaging, especially in early-stage cancer and disease diagnosis.^5–10^ At these ultrasmall dimensions, strong quantum confinement causes the continuous electronic density of states to break into discrete energy levels, resulting in size-dependent fluorescence and conferring molecule-like properties such as versatile surface functionalization and tunable emission.^11–13^

Compared with fluorescence in the ultraviolet (UV: <400 nm) or visible (VIS: 400–700 nm) regions, near-infrared (NIR: 700–1800 nm) emission offers distinct advantages for bioimaging.^14^ The reduced tissue autofluorescence and lower scattering in the NIR window enable higher imaging sensitivity and deeper tissue penetration.^15–17^

AuNCs are commonly synthesized by reducing Au^3+^ to Au^+^ species in the presence of both reducing and capping agents.^18^ Thiol-containing ligands are widely used because of their strong Au–S bonds that stabilize the clusters and facilitate the formation of well-defined Au-thiolate motifs.^19,20^ Among these ligands, glutathione (GSH), a tripeptide composed of glycine, cysteine, and glutamic acid, has been extensively employed as both the reducing agent and surface ligand for preparing GSH-AuNCs.^21^ The optical properties of GSH-AuNCs are highly sensitive to their atomic structure and surface chemistry.^22,23^

Small variations in synthesis parameters, including the pH, temperature, and ligand-to-metal ratio, can significantly influence the Au­(I)/Au(0) balance, staple-motif configuration, and ligand packing density, resulting in substantial changes in the emission wavelength and intensity of GSH-AuNCs.^24,25^ Although previous studies have demonstrated that individual or paired synthetic parameters can regulate the optical properties of gold nanoclusters, the combined effects of these parameters on the transition between visible and NIR emission states and the underlying structural origins of this evolution remain insufficiently understood. Establishing such structure–property relationships is essential for the rational design of GSH-AuNCs with tailored optical properties for advanced applications.^26^

In this work, we systematically investigate the formation of NIR-emitting GSH-AuNCs by regulating the reaction pH, temperature, and gold precursor concentration. Beyond identifying the synthetic conditions required for strong NIR emission, we correlate fluorescence behavior with structural, chemical, and excited-state characteristics using complementary spectroscopic and scattering analyses. This integrated approach provides mechanistic insight into how synthesis conditions govern gold core growth, Au­(I)-thiolate ligand shell organization, and electronic state evolution, enabling rational control over the emission properties of GSH-AuNCs.

To demonstrate the potential of the NIR-emitting GSH-AuNCs for targeted biosensing, both referenced red-emissive and NIR-emissive GSH-AuNCs were functionalized with the SYL3C aptamer, a SELEX-derived oligonucleotide that selectively recognizes the epithelial cell adhesion molecule (EpCAM), a biomarker overexpressed in numerous epithelial cancers.^27–31^ The targeting performance of the resulting SYL3C-GSH-AuNC nanoprobe was evaluated by flow cytometry using EpCAM-positive PC3 prostate cancer cells and EpCAM-negative HEK293 cells.^32–35^ The functionalized NIR-emissive nanoprobes exhibited selective binding toward EpCAM-expressing cells, demonstrating their promise as biocompatible targeting imaging probes with reduced background autofluorescence and enhanced contrast for cancer bioimaging.

## 2 Materials and Methods

### 2.1 Materials

Hydrogen tetrachloroaurate trihydrate (HAuCl_4_·3H_2_O), sodium hydroxide (NaOH), and l-glutathione (GSH) were obtained from Sigma-Aldrich. Phosphate-buffered saline (PBS) was provided by Biochemical Reagents. Sodium phosphate monobasic, sodium phosphate dibasic, tris­(2-carboxyethyl) phosphine hydrochloride (TCEP), TE buffer solution pH 8.0, methanol, ethanol, sodium chloride (NaCl), Dulbecco’s Modified Eagle Medium (DMEM), fetal bovine serum (FBS), penicillin–streptomycin, and sodium dodecyl sulfate (SDS) were purchased from Sigma-Aldrich. l-Glutamine was obtained from VMR. 5. Gibco TrypLE Express Enzyme (1×) was purchased from Fisher Scientific.

### 2.2 Characterization

Ultraviolet–visible (UV–vis) absorption spectra were taken using a PerkinElmer Lambda 25 UV/vis spectrometer from 265 to 600 nm with a 2 nm bandwidth.

Fluorescence emission spectra and the excitation–emission matrix (EEM) were measured using a Horiba Fluorolog 3 with a 3.5 nm bandwidth. Photoluminescence quantum yield (PLQY) measurements were performed by using an FLS1000 spectrometer (Edinburgh Instruments) equipped with a visible PMT-980 detector and an integrating sphere.

Time-correlated single photon counting (TCSPC) measurements were performed through a Horiba Jobin Yvon Fluorolog-3 equipped with the 374 nm DeltaDiode laser source at the 50 kHz repetition rate. Data analysis was performed using the IBH DAS6 software. The fluorescence intensity decay curves were fitted using a multiexponential decay model, expressed as

1 $$I\left(t\right)=\Sigma_{i}\alpha_{i}\exp\left(-\frac{t}{\tau_{i}}\right)$$

where τ_*i*_ represents the fluorescence lifetime of the *i*th decay component, and α_*i*_ is its corresponding pre-exponential amplitude.

The intensity-weighted average lifetime (τ̅) is calculated as

2 $$\mathrm{\tau ̅}=\frac{\sum_{i}\alpha_{i}\tau_{i}^{2}}{\sum_{i}\alpha_{i}\tau_{i}}$$

A Malvern Zetasizer Nano ZS instrument was used to measure the zeta potential of nanoparticles using the laser Doppler velocimetry (LDV) technique, and the value was calculated using Henry’s equation

3 $$\zeta =\frac{3\eta \mu_{e}}{2\varepsilon f\left(\kappa \alpha \right)}$$

where ζ is the zeta potential, ε is the dielectric constant of water, η is the solution viscosity, and *f*(κα) is the function of the dimensionless parameter κα.

Circular dichroism (CD) spectra were recorded using a Jasco J-1500 spectropolarimeter. Far-UV CD spectra were collected in 10 mm path-length quartz cuvettes (Hellma Analytics, Southend-on-Sea, UK) at 25 °C in the 180–450 nm range, at a scanning speed of 100 nm/min, a response time of 1s, a bandwidth of 2 nm, and a data pitch of 0.1 nm.

The pH value was measured using an Accumet AE150 pH meter with an AE series double junction Ger pH electrode from Fisher Scientific.

X-ray photoelectron spectroscopy (XPS) was performed using a monochromatic Al Κα source (*h*ν = 1486 eV) through Kratos AXIS Supra+. Prior to XPS measurements, the GSH-AuNC dispersion was solvent-exchanged to diH_2_O. All measurements employed charge neutralization using low-energy electrons to compensate for surface charging. Peak fitting and data analysis were carried out using KherveFitting software.^36^

Small-angle X-ray scattering (SAXS) sample preparation and measurement conditions were based on the protocol described previously.^37^ SAXS measurements were performed using a Xenocs Xeuss 2.0 SAXS/WAXS instrument (Xenocs, Grenoble, France) equipped with a Genix3D microfocus Cu Kα1 microfocus X-ray source (l = 1.5406 Å) and a DECTRIS Pilatus 1 M detector. Data analysis was performed using the BioXTAS RAW software.^38^

### 2.3 Synthesis of Glutathione-Capped Gold Nanoclusters

The glutathione-capped gold nanoclusters (GSH-AuNCs) were synthesized following a reported method^39^ with modified synthesis conditions (detailed below). Briefly, an aqueous solution of HAuCl_4_ (20 mM, 0.5 mL) was mixed with GSH (100 mM, 0.15 mL) and diluted with 4.35 mL of distilled water (diH_2_O) at room temperature. The mixture was then heated to 70 °C with shaking at 500 rpm for 24 h. The experiment temperature and shaking speed were controlled by the ThermoMixer C from Eppendorf. The resulting GSH-AuNCs were stored at 4 °C in the dark, and no significant changes in their optical properties were observed over 6 months. Prior to optical measurements, 300 μL of GSH-AuNCs was diluted to 2.7 mL with 0.01 M PBS buffer (pH 7.2–7.6) and equilibrated for 10 min.

#### 2.3.1 Synthesis of NIR GSH-AuNCs at Different Concentrations of HAuCl_4_ (*C*_(Au)_)

0.5 mL of aqueous solutions of HAuCl_4_ at concentrations from 20 mM to 40 mM in 2 mM increments was mixed with GSH (100 mM, 0.15 mL) and diluted to 4.35 mL with distilled water (diH_2_O) at room temperature. The mixture was then heated to 70 °C with shaking at 500 rpm for 24 h.

#### 2.3.2 Synthesis of NIR GSH-AuNCs at Different NaOH Volumes

The reaction pH was systematically varied by adding 1 M NaOH to the aqueous mixture of HAuCl_4_ and GSH before heating and shaking. The volume of NaOH was increased incrementally from 20 to 90 μL in 5 μL increments.

#### 2.3.3 Synthesis of NIR GSH-AuNCs at Different Reaction Temperatures

GSH-AuNCs were synthesized at 70 °C, 80 °C, 90 °C, and 95 °C in combination with varying reaction pH or *C*_(Au)_ manipulation.

### 2.4 Functionalization of GSH-AuNCs with the SYL3C-Aptamer

As-made, red- and NIR-emissive GSH-AuNCs were purified by precipitation with 3 volumes of methanol, followed by centrifugation at 13,000 rpm for 15 min. The resulting pellet was redispersed in 100 μL of diH_2_O to a final concentration of 40 μM.

Prior to functionalization, the thiol-modified SYL3C aptamer was reduced with TCEP to generate free thiol groups. The reduced aptamer was then mixed with red- or NIR-emissive GSH-AuNCs at an aptamer-to-cluster molar ratio of 2.5:1. Conjugation was performed using the salt aging method as previously described.^40,41^ The resulting GSH-AuNC–aptamer conjugates were purified by dialysis (20 kDa molecular weight cutoff) against PBS at 4 °C, with the buffer replaced every 2 h for three changes, followed by overnight dialysis.

### 2.5 Flow Cytometry Analysis of GSH-AuNCs–SYL3C–Aptamer Nanoprobes

EpCAM-positive PC3 and EpCAM-negative HEK293 cells were cultured as previously described.^42^ Cells were incubated with nanoprobes at a final concentration of 50 nM for 4 h, washed five times with PBS to remove unbound probes, and resuspended in 500 μL of ice-cold PBS for flow cytometric analysis.

Flow cytometry was performed using an Attune NXT Flow Cytometer (Thermo Fisher Scientific) with a 488 nm blue laser (50 mV beam-shaping optics and 100 mV diode power). Red-emissive nanoprobes were detected using the BL3 channel (695/40 nm emission), while NIR-emissive nanoprobes were analyzed using the BL4 channel (780/60 nm emission).

## 3 Results and Discussion

### 3.1 Impact of the Single Growth Parameter on the Fluorescence of NIR GSH-AuNCs

#### 3.1.1 Impact of Reaction pH

Reaction pH was systematically adjusted by adding varying volumes of 1 M NaOH (20–90 μL in 5 μL intervals), resulting in pronounced changes in the fluorescence intensity of GSH-AuNCs at 640 and 825 nm (NIR). Digital photographs of the synthesized GSH-AuNCs under visible and UV illumination are presented in Figure 1a,b, respectively, while the correlation between NaOH volume, *V*_(NaOH),_ and the initial mixture pH is shown in Figure 1c. Under acidic synthesis conditions, the GSH-AuNCs exhibited strong red fluorescence upon UV excitation. In contrast, fluorescence was nearly quenched when 55 μL (pH 5.15) or 60 μL (pH 5.99) of NaOH was added during synthesis. Although weak fluorescence signals persisted under alkaline conditions, the emission intensity was substantially lower than that observed in the acidic environment.

**Figure 1:**
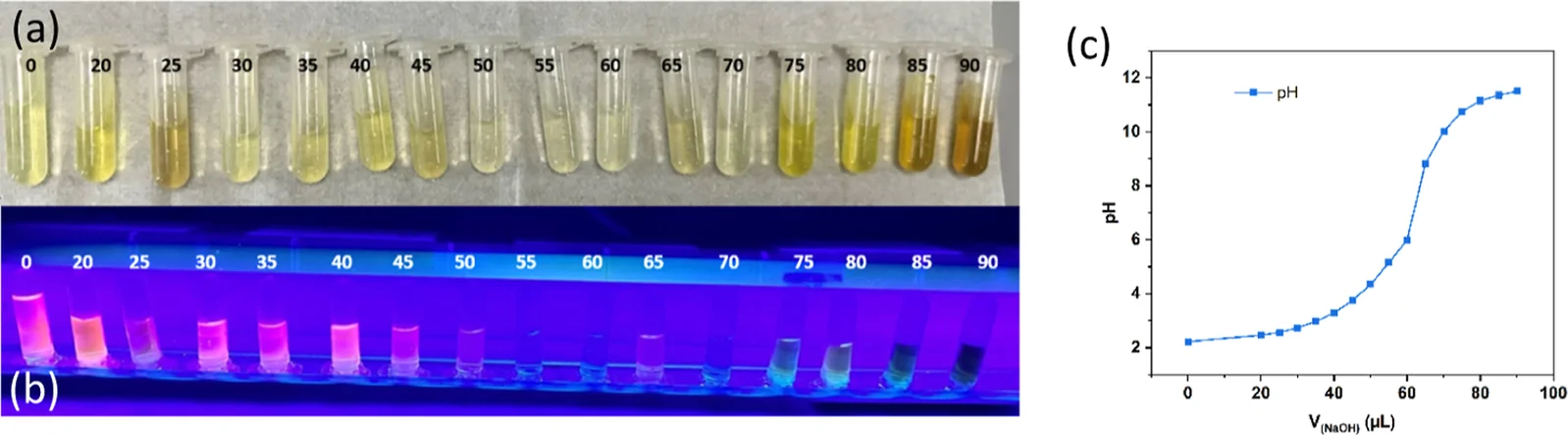
GSH-AuNCs synthesized by adding different *V*_(NaOH)_, (a) under white light and (b) under UV light. (c) Relationship between *V*_(NaOH)_ and pH in the GSH and HAuCl_4_ mixture.

To investigate the photophysical properties of the synthesized GSH-AuNCs, UV–vis absorption spectra were recorded, and the absorbance at 265 nm was compared, as summarized in Figure 2. Samples prepared with 0–40 μL of 1 M NaOH exhibited a broad absorption band centered around 400 nm, with the absorption intensity gradually decreasing as the pH increased. For samples synthesized with 45–90 μL of 1 M NaOH, the main absorption band shifted to approximately 375 nm, except for the sample prepared with 55 μL of NaOH. This sample showed lower overall absorption and two distinct UV–vis absorption peaks at 330 and 375 nm, consistent with the reported Au_10–12_SG_10–12_ species.^43^ Notably, no band at 520 nm, characteristic of the surface-plasmon resonance of spherical gold nanoparticles larger than 5 nm, was observed in any of the samples, confirming the absence of larger gold nanoparticles.

**Figure 2:**
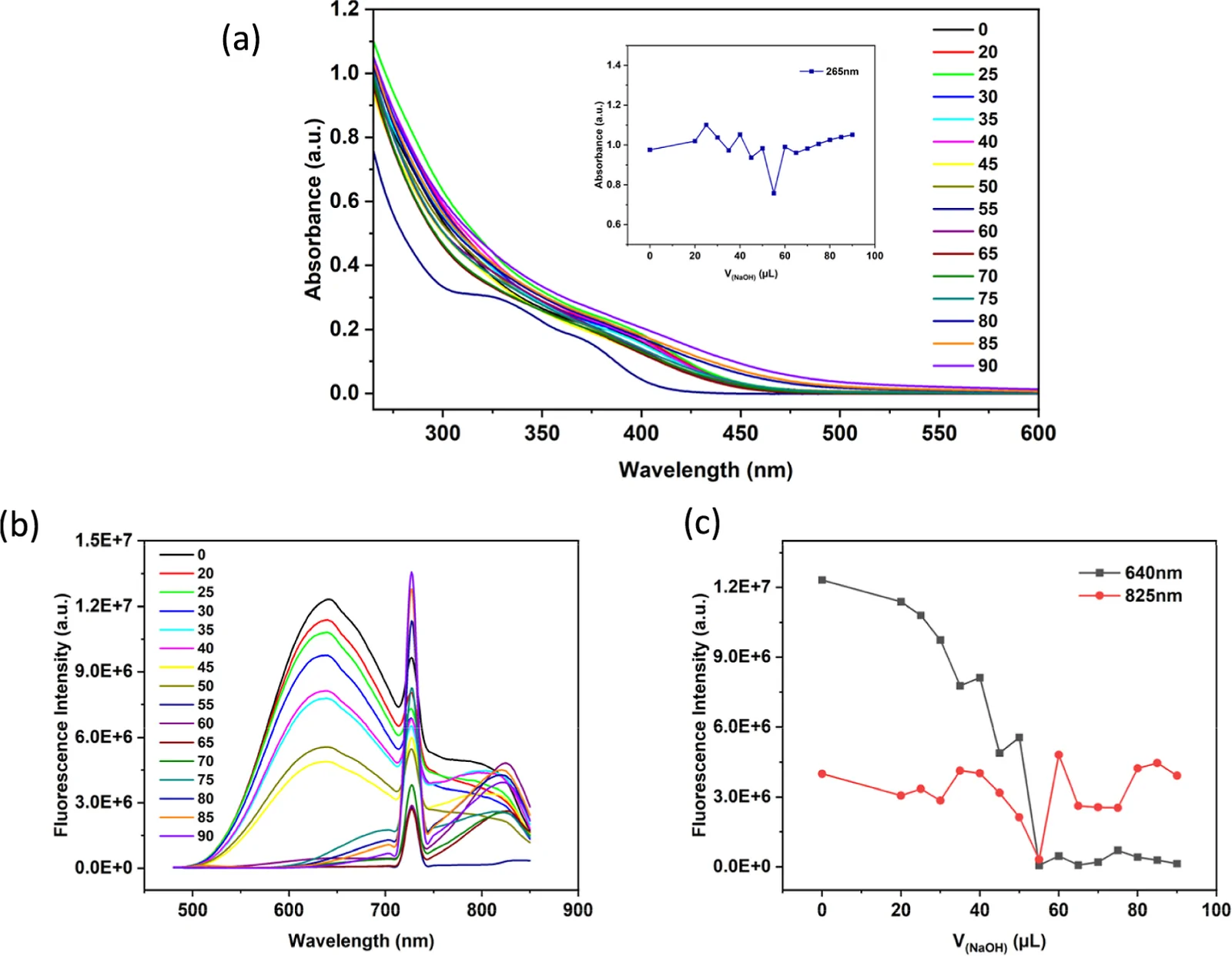
(a) UV–vis absorption spectra of GSH-AuNCs synthesized with various *V*_(NaOH),_ and the inset showing the corresponding intensity of absorption at 265 nm. (b) Fluorescence emission spectra of the same samples under 365 nm excitation; (c) fluorescence intensities of 640 and 825 nm emissions.

Figure 2b presents the fluorescence emission spectra under 365 nm excitation; the sharp peaks centered at 730 nm observed were attributed to second-order diffraction through the monochromator. Samples prepared with 0–50 μL of 1 M NaOH exhibited a strong emission band centered at 640 nm. As shown in Figure 2c, the intensity of the 640 nm emission gradually decreased with increasing *V*_(NaOH)_ and disappeared when *V*_(NaOH)_ exceeded 55 μL, while the emission at 825 nm remained relatively constant. These results indicate that varying the pH alone does not promote the formation of nanoclusters with NIR emission. Notably, the sample synthesized with 55 μL of 1 M NaOH showed no detectable fluorescence in this spectral range.

#### 3.1.2 Impact of HAuCl_4_ Concentration *C*_(Au)_

Figure 3a shows the fluorescence spectra of GSH-AuNCs synthesized with varying HAuCl_4_ concentrations, *C*_(Au)_, ranging from 20 mM to 40 mM in 2 mM increments without adding NaOH. The peak emission intensities at 640 and 825 nm are compared in Figure 3b. The 640 nm emission decreases with an increase in *C*_(Au)_ and disappears when *C*_(Au)_ exceeds 36 mM. In contrast, the NIR emission at 825 nm gradually increased with *C*_(Au)_, reaching a maximum at 34 mM before decreasing at higher concentrations. These results indicate that varying the HAuCl_4_ concentration modulates the emission behavior of GSH-AuNCs, shifting from a single dominant emission centered at 640 nm to dual emissions at 640 and 825 nm and ultimately to a single dominant NIR emission centered at 825 nm.

**Figure 3:**
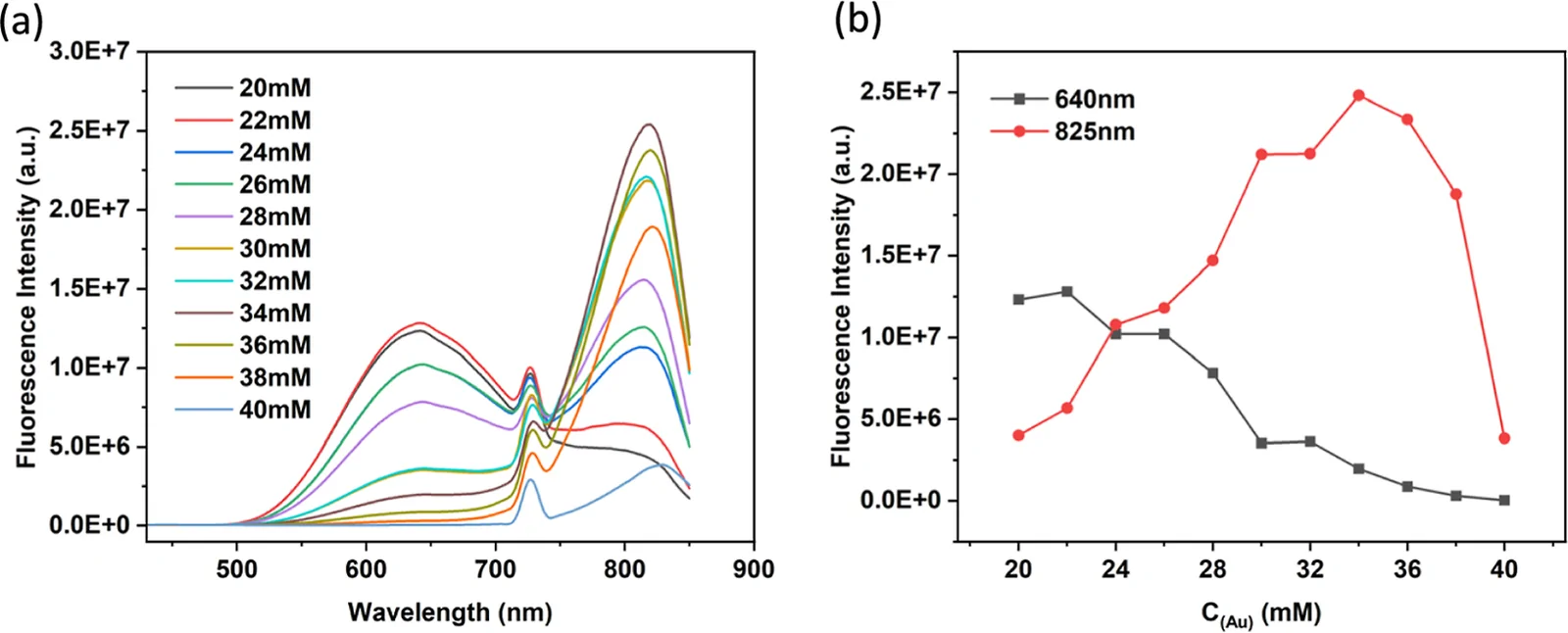
(a) Emission spectra of the GSH-AuNCs synthesized at different *C*_(Au)_, excited at 365 nm, and (b) fluorescence intensity comparison of 640 and 825 nm emissions.

### 3.2 Double Parameters Modified to Investigate NIR GSH-AuNC Generation

To further enhance the NIR emission, the combined effects of reaction pH, temperature, and *C*_(Au)_ were investigated. Samples were synthesized at temperatures of 70 °C, 80 °C, 90 °C, and 95 °C. Within each temperature set, either the reaction pH or *C*_(Au)_ was systematically varied to evaluate the simultaneous influence of these three parameters on the NIR fluorescence properties of the GSH-AuNCs.

#### 3.2.1 Impact of Reaction Temperatures and pH

The fluorescence intensity of the dual-emission peaks of GSH–AuNCs synthesized at 70 °C, 80 °C, 90 °C, and 95 °C with varying *V*_(NaOH)_ under 365 nm excitation is summarized in Figure 4. Overall, the fluorescence intensity of the 640 nm emission decreased with increasing *V*_(NaOH)_ and nearly disappeared when more than 55 μL of 1 M NaOH was added (mixture pH > 5.15). Higher reaction temperature further suppressed the 640 nm emission. In contrast, the NIR emission at 825 nm remained relatively stable up to 45 μL of NaOH across all temperature groups, followed by a decline in the 50–55 μL range. Samples synthesized at higher temperatures exhibited comparatively stronger NIR emission. Notably, sharp increases in fluorescence intensity were observed for samples prepared at 90 and 95 °C, with maxima occurring at *V*_(NaOH)_ ≈ 60–70 μL, before decreasing again when *V*_(NaOH)_ exceeded 75 μL (pH > 10.76). These results indicate that near-neutral pH and elevated temperatures significantly enhance the yield of NIR-emitting GSH-AuNCs.

**Figure 4:**
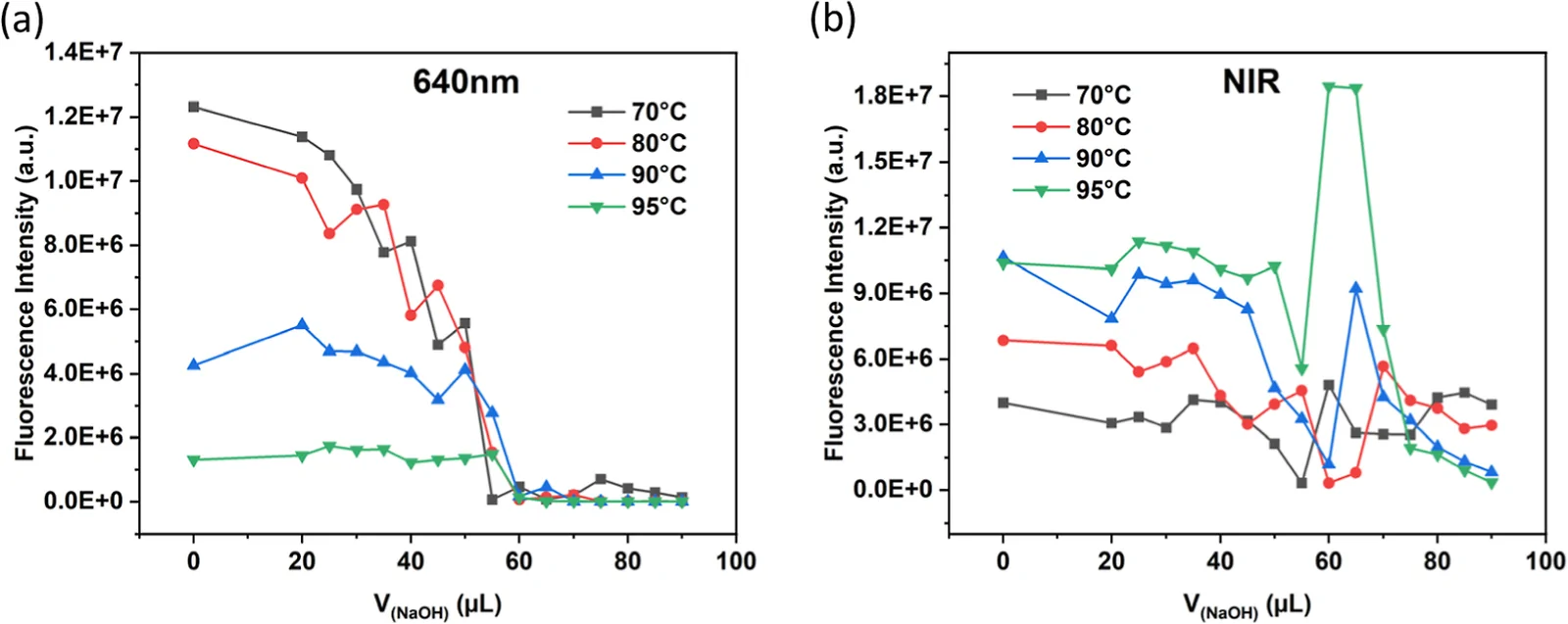
GSH-AuNCs fluorescence intensity comparison at 640 nm (a) and 825 nm (b) emission (λ_ex_ = 365 nm), synthesized under various reaction temperatures and *V*_(NaOH)._

#### 3.2.2 Impact of Reaction Temperatures and *C*_(Au)_

Figure 5 compares the fluorescence intensities at 640 and 825 nm for GSH-AuNCs synthesized at 70 °C, 80 °C, 90 °C, and 95 °C with varying HAuCl_4_ concentrations, *C*_(Au)_, ranging from 20 mM to 40 mM in 2 mM increments under 365 nm excitation without adding NaOH. The intensity of the 640 nm emission decreased with increasing *C*_(Au)_ and temperature, eventually becoming nearly undetectable. In contrast, the NIR emission at 825 nm initially increased with *C*_(Au)_, reached a maximum, and then declined at higher concentrations. Elevated temperatures enhanced the NIR emission at lower *C*_(Au)_ (<26 mM); however, at higher *C*_(Au)_ (32 mM–40 mM), samples prepared at 70 and 80 °C exhibited stronger NIR emission. The optimal *C*_(Au)_ for maximum NIR emission depended on the reaction temperatures, occurring at 34 mM at 70 and 80 °C, 28 mM at 90 °C, and 26 mM at 95 °C.

**Figure 5:**
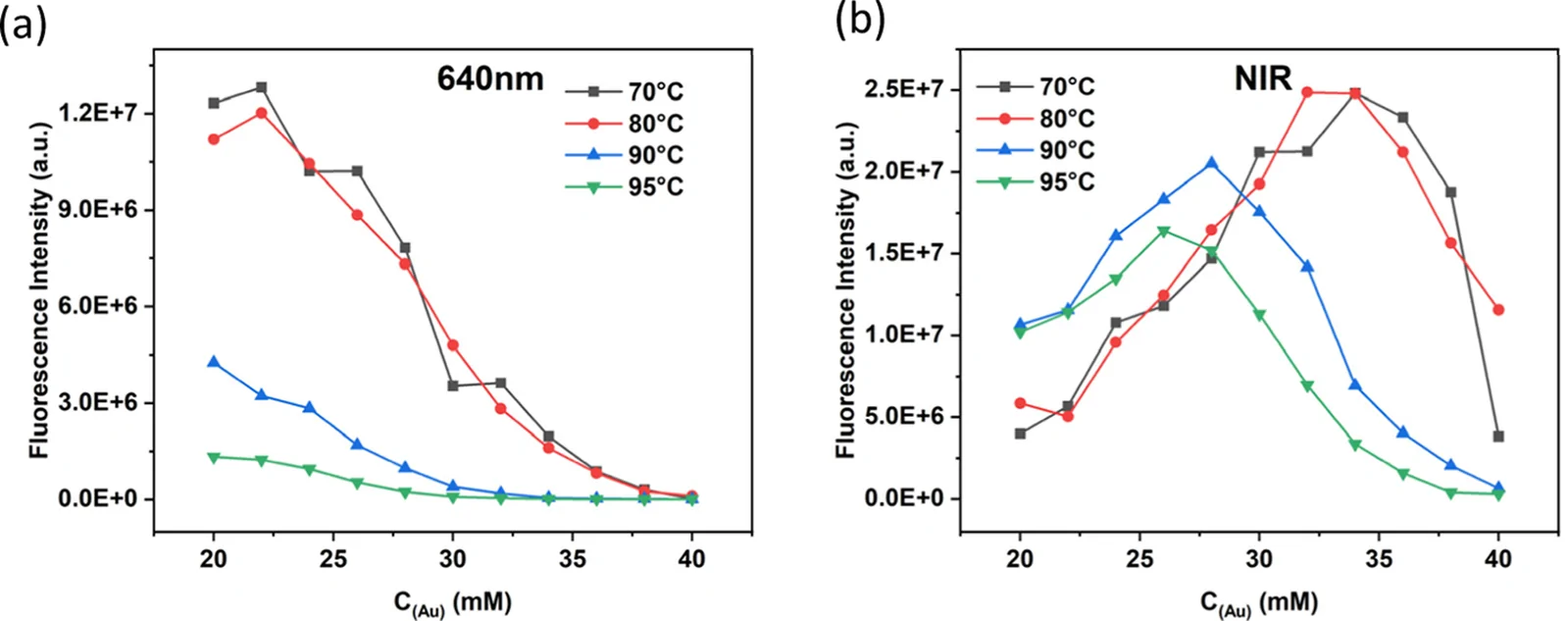
GSH-AuNCs’ fluorescence intensity comparison at 640 nm (a) and 825 nm (b) emission (λ_ex_ = 365 nm) synthesized at various reaction temperatures and *C*_(Au)_.

### 3.3 Tuning of the Optimal Growth of NIR GSH-AuNCs

Compared with the strongest NIR emissions obtained by varying pH and temperature, modulation of *C*_(Au)_ and temperature produced GSH-AuNCs with higher NIR emission. To further enhance the NIR intensity, the synthesis conditions were fine-tuned by varying pH at the optimal temperature—*C*_(Au)_ combinations, specifically, 34 mM at 70 and 80 °C, 28 mM at 90 °C, and 26 mM at 95 °C.

The NIR fluorescence intensities as a function of *V*_(NaOH)_, *C*_(Au),_ and temperature are summarized in a 3D plot (Figure 6a). The red star marks the optimal synthesis condition identified in this study: 95 °C, *C*_(Au)_ = 26 mM, and 80 μL of 1 M NaOH, which yielded the strongest NIR fluorescence. The second strongest sample was formed at 90 °C, *C*_(Au)_ = 28 mM, and 75 μL of 1 M NaOH. Figure 6 compares the UV–vis absorption and fluorescence emission spectra of GSH-AuNCs prepared under the optimal conditions with those synthesized using a reported method.^39^ Both samples exhibited similar absorption profiles; however, the optimal NIR sample displayed a red shift, with the absorption tail extending to approximately 500 nm. Notably, the optimal sample (red curve) showed higher absorbance than the reference sample (black curve), suggesting enhanced formation of NIR-emitting GSH-AuNCs under the optimal reaction conditions. After absorption correction, the NIR emission at 825 nm of the optimal samples was approximately 7.2 times stronger than the red emission of the reference sample. The absolute photoluminescence quantum yield (PLQY) for the reference and optimal samples was measured as 1.14% and 5.64%, respectively, indicating that the optimal NIR GSH-AuNCs obtain more efficient fluorescence and stronger signals in imaging and sensing applications.

**Figure 6:**
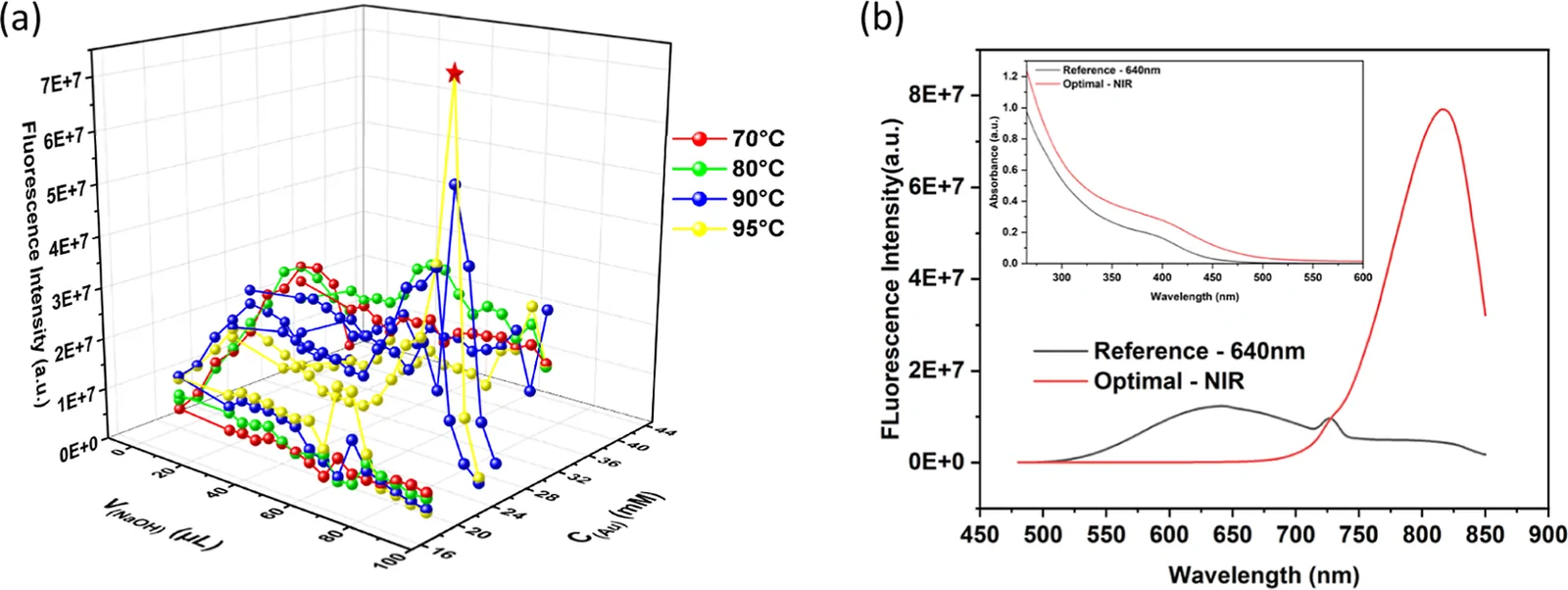
(a) 3D scatter plot of fluorescence intensity of GSH-AuNCs synthesized under manipulation of reaction conditions (*V*_(NaOH)_, *C*_(Au),_ and temperature). (b) Fluorescence spectra of GSH-AuNCs prepared under the optimal synthesis condition (red curve) and the reported method^39^ (black curve); the inset shows the corresponding UV–vis absorption spectrum.

The formation of GSH-AuNCs proceeds through a sequential Au­(III) to Au­(I) to Au(0) pathway. In this process, thiol-mediated reduction of Au­(III) generates stable Au­(I)–thiolate complexes, which kinetically suppress uncontrolled growth into larger nanoparticles. These Au­(I)–thiolate intermediates act as key precursors that regulate the nucleation of ultrasmall Au(0) gold cores, producing molecular-sized nanoclusters with discrete electronic states and size-dependent fluorescence, including NIR emission. Owing to the high stability, specific coordination structure, and redox behavior of Au­(I)–thiolate complexes, their formation and evolution are highly sensitive to reaction conditions. Consequently, parameters such as the GSH/gold ratio, reaction pH, and temperature play critical roles in governing nanocluster formation and emission characteristics.

The pH-dependent dual emission of GSH-AuNCs observed in this study is likely to originate from protonation-controlled ligand conformation and surface rigidity. At low pH, neutral –COOH groups enable compact GSH packing and stabilize small, rigid clusters, providing the conditions for strong, shorter emission at 640 nm. Progressive deprotonation of –COOH groups between pH 3.3 and 5.5 introduces electrostatic repulsion, destabilizing both 640 nm and NIR-emissive structures and leading to complete fluorescence quenching. At near-neutral pH, extended GSH is likely to form larger or more delocalized Au­(I)-rich structures, with the lack of effective luminescent centers of GSH-AuNCs,^44^ leading to the sudden emergence and stabilization of NIR emission, while the 640 nm emitted GSH-AuNCs are hard to generate.

The reaction temperature strongly affects the nucleation and growth kinetics of the gold core during GSH-AuNC formation. At elevated temperatures, the reduction of Au^3+^ to Au^0^ proceeds more rapidly, generating a burst of nuclei, followed by accelerated atom diffusion and core growth. Faster thermal atomic migration also promotes relaxation of the metal core. As the gold core grows and becomes more ordered, its electronic energy levels become more closely spaced; the increased particle size generally weakens the quantum confinement effect and results in the red shift of the emission peak of clusters.^45,46^

Figure 6a indicates that the growth of NIR-emissive GSH-AuNC is a highly intricate and sensitive process, governed by the combined effects of the GSH/gold ratio, reaction pH, and temperature. Optimal nanocluster formation occurs within a well-defined parameter window.

### 3.4 Evolution of Fluorescence Properties and Surface Characteristics of GSH-AuNCs

With respect to fluorescence behavior, three distinct emission profiles of GSH-AuNCs were identified under the synthesis conditions investigated in this study. Figure 7a presents the emission spectra of representative samples synthesized at 70 °C without NaOH using different HAuCl_4_ concentrations, *C*_(Au)_ = 20 mM, 28 mM, and 34 mM. The reference sample prepared at 20 mM exhibited a dominant single emission centered at 640 nm. At 28 mM, two emission bands were observed, with the NIR emission surpassing the intensity of the 640 nm band. Increasing *C*_(Au)_ to 34 mM resulted in a spectrum dominated by NIR emission, with an intensity approximately 2.5 times greater than the 640 nm emission of the reference sample. The quantum yields of red-, dual-, and NIR-emissive clusters were found to be 1.14% (640 nm), 2.08% (825 nm), and 2.31% (825 nm), respectively.

**Figure 7:**
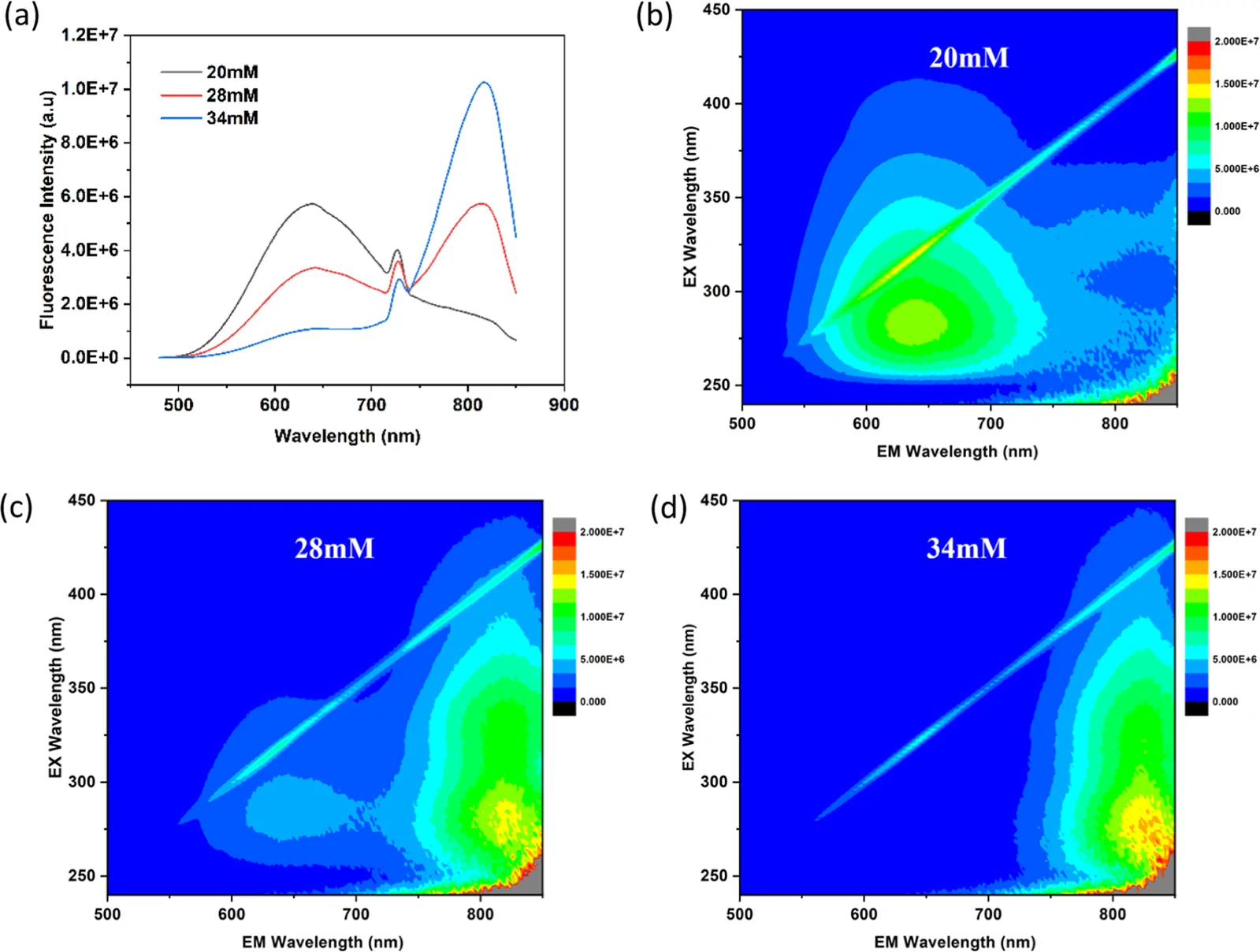
(a) Emission spectra (λ_ex_ = 365 nm) of GSH-AuNCs synthesized under different *C*_(Au)_ and corresponding EEM with λ_ex_ from 300 to 500 nm and λ_em_ from 500 to 850 nm. (b) *C*_(Au)_ = 34 mM, (c) *C*_(Au)_ = 20 mM, and (d) *C*_(Au)_ = 28 mM.

The excitation–emission matrices shown in Figure 7b–d further illustrate the evolution of fluorescence properties. The NIR GSH-AuNCs exhibit a broad excitation window spanning 250–420 nm, with a maximum excitation around 275 nm, consistent with ligand- and metal-centered electronic transitions commonly observed in ultrasmall gold nanoclusters.

Fluorescence lifetime analysis results of the above three clusters are summarized in Tables 1 and 2 for the 640 and 800 nm emission bands, respectively. The fluorescence decay curves were analyzed using a 4-exponential model, with one fixed component (τ_4_ = 1.66 ns) accounting for scattering. All samples exhibited three lifetime components in the ranges of tens of nanoseconds, hundreds of nanoseconds, and microseconds, consistent with the multiple emissive states and relaxation pathways commonly observed in thiolate-protected gold nanoclusters.^47^

**Table 1:** Fluorescence Lifetime Fitted Data for GSH-AuNCs Synthesized under Various *C*_(Au)_ (λ_ex_ = 374 nm, λ_em_ = 640 nm)

*C*_(Au)_/mM	τ_1_/ns	Β_1_/%	τ_2_/ns	Β_2_/%	τ_3_/ns	Β_3_/%	τ_4_/ns	Β_4_/%	χ^2^	τ̅/ns
20	3244 ± 48	65.73	765 ± 36	28.55	122 ± 6	5.33	1.66	0.39	1.10	3006 ± 44
28	2994 ± 48	60.96	669 ± 42	31.28	108 ± 6	7.06	1.66	0.69	1.17	2745 ± 43

**Table 2:** Fluorescence Lifetime Fitted Data for GSH-AuNCs Synthesized under Various *C*_(Au)_ (λ_ex_ = 374 nm, λ_em_ = 800 nm)

*C*_(Au)_/mM	τ_1_/ns	Β_1_/%	τ_2_/ns	Β_2_/%	τ_3_/ns	Β_3_/%	τ_4_/ns	Β_4_/%	χ^2^	τ̅/ns
28	3476 ± 132	82.83	684 ± 90	15.03	51 ± 15	1.46	1.66	0.68	1.17	3379 ± 111
34	3655 ± 138	78.12	1048 ± 201	20.28	105 ± 15	1.38	1.66	0.21	1.12	3473 ± 129

The longest component (τ_1_) represents the dominant emissive state and is commonly associated with ligand–metal charge-transfer (LMCT) character and metal-centered excited states influenced by the strong spin–orbit coupling of gold atoms. The intermediate component (τ_2_) may arise from additional charge-transfer states, including ligand-to-metal–metal charge-transfer (LMMCT) states involving Au­(I)–Au­(I) interactions. The shortest component (τ_3_) is attributed to rapidly decaying emissive populations associated with less stabilized excited states or heterogeneous emissive environments within the nanocluster structure.^48,49^

Comparison of the fitted lifetime parameters reveals that changes in gold precursor concentration modify the excited-state dynamics while preserving similar overall decay characteristics. At 640 nm emission, the red-emissive GSH-AuNCs exhibited a longer dominant lifetime (τ_1_ = 3244 ns; τ̅ = 3006 ns) compared with the dual-emissive sample (τ_1_ = 2994 ns; τ̅ = 2745 ns). In contrast, for 800 nm emission, the NIR-emissive clusters showed a modest increase in lifetime (τ_1_ = 3655 ns; τ̅ = 3473 ns) compared with the dual-emissive clusters (τ_1_ = 3476 ns; τ̅ = 3379 ns). These results indicate that the emergence of NIR emission is accompanied by subtle changes in excited-state stabilization, although the lifetime variation does not follow a simple monotonic relationship with emission wavelength.

The slightly prolonged lifetime observed for NIR-emissive GSH-AuNCs suggests a greater contribution from long-lived emissive states, potentially arising from changes in cluster nuclearity and electronic coupling between the gold core and ligand shell. Such modifications can influence the relative contribution of different charge-transfer states and alter the balance between radiative and nonradiative relaxation pathways. The similar multiexponential decay profiles across all samples indicate that the fundamental excited-state relaxation mechanism remains comparable, while synthetic conditions tune the relative population and energy of emissive states responsible for the observed emission shift^50^.

Figure 8a compares the circular dichroism (CD) spectra of the three GSH-AuNC samples with that of free l-GSH. Free l-GSH displays a single negative band centered at ∼210 nm and no discernible features above 250 nm, consistent with the CD signature of a nonaromatic tripeptide composed of l-Glu, l-Cys, and Gly in a predominantly random-coil conformation.^51^ In contrast, all three GSH-AuNC samples exhibit positive CD bands around 210 nm, indicative of induced chirality arising from ligand coordination and ordered assembly within the chiral Au­(I)–thiolate shell. Additionally, two induced CD bands appear in the 250–320 nm region (band I) and 350–400 nm (band II), which are commonly attributed to LMCT, LMMCT, and Au­(I)–thiolate staple or shell-related electronic transitions.^52^ Notably, band I in the dual-emissive and NIR-emissive samples is blue-shifted relative to that of the red-emissive sample, while band II in the NIR-emissive sample shows a further blue shift compared to the other two. These spectral changes suggest that increasing gold precursor concentration alters the organization and electronic structure of the ligand shell. Although the overall absorption profiles remain similar, the CD spectral evolution reveals subtle changes in chiral metal–ligand interactions that can influence the distribution and relaxation of excited states, contributing to the observed emission transition from 640 to 825 nm. The intermediate CD features and dual emission observed at 28 mM further suggest that this sample represents a transitional electronic structure rather than a physical mixture of red- and NIR-emissive species.

**Figure 8:**
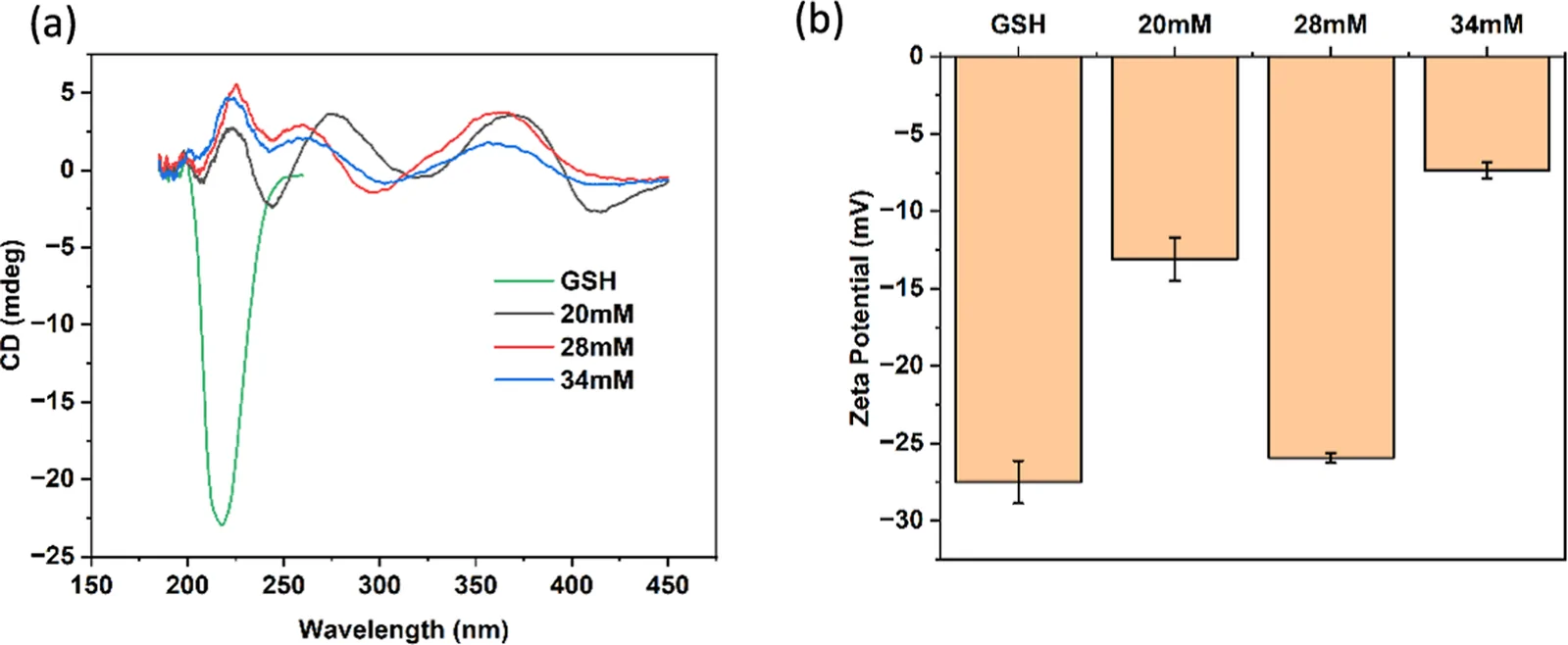
(a) CD spectra and (b) zeta potentials for l-GSH only and l-GSH-AuNCs synthesized under different *C*_(Au)_.

The surface charge characteristics of GSH-AuNCs were further investigated by zeta potential measurements in 0.01 M PBS (pH 7.4) (Figure 8b). Free GSH exhibited a ξ-potential of −27.50 ± 1.38 mV, consistent with deprotonation of its two carboxyl groups and protonation of the amine at physiological pH, yielding an overall negative charge. In contrast, GSH-AuNCs synthesized at *C*_(Au)_ = 20 mM, 28 mM, and 34 mM showed zeta potentials of −13.10 ± 1.40 mV, −25.93 ± 0.31 mV, and −7.34 ± 0.52 mV, respectively. These variations indicate that cluster formation does not simply reduce surface charge but induces concentration-dependent reorganization of the GSH ligand shell. The less negative potential observed for 20 mM and 34 mM samples suggests increased charge shielding associated with Au-ligand coordination and altered ligand orientation, while the 28 mM sample retains a surface potential comparable to free GSH, indicating greater exposure of ionized functional groups.

Such differences are consistent with dynamic coordination behavior of GSH on gold nanoclusters. Previous molecular dynamics studies have suggested that GSH preferentially coordinates through the cysteine thiol group, while the peptide backbone and terminal functional groups adopt different orientations depending on surface accessibility and ligand density.^34^ Changes in ligand packing, peptide conformation, and hydration environment can therefore alter the effective surface charge measured by ζ-potential. The distinct surface charge characteristics of the three samples support the presence of different ligand-shell organization rather than a simple correlation with gold content.

The densities of free GSH and the three GSH-AuNCs were also measured at 600 ppm. The 28 mM sample exhibited the highest density (1.0046 g/cm^3^), followed by 20 mM (1.0045 g/cm^3^), 34 mM (1.00445 g/cm^3^), and free GSH (1.00435 g/cm^3^). Although the absolute differences are small, the reproducible increase relative to free GSH indicates incorporation of the high-density gold component and suggests subtle variations in the metal-to-ligand ratio, structural compactness, and/or solvation among the nanocluster formulations.

Combining the fluorescence, CD, ζ-potential, and density measurements reveals a complex structure–property relationship. The emission red shift from 640 nm (20 mM) to 825 nm (34 mM) is accompanied by changes in ligand-shell organization and surface electrostatics rather than arising solely from gold core growth. Notably, the 28 mM dual-emissive sample exhibits the highest measured density while maintaining a relatively negative surface potential, suggesting that an increased structural compactness does not necessarily correspond to enhanced charge shielding. These observations indicate that metal core evolution and ligand shell reorganization occur in a partially decoupled manner.

X-ray photoelectron spectroscopy (XPS) was subsequently employed to investigate the chemical composition and oxidation status of differently emissive GSH-AuNCs. Similar spectral features were observed for all samples; representative spectra of the NIR-emissive GSH-AuNCs are shown in Figure 9. The Au 4f spectrum exhibited characteristic doublet peaks at 84.10 and 87.87 eV, corresponding primarily to Au(0).^39^ The S 2p spectrum displayed peaks at 162.55 and 163.75 eV, corresponding to Au–S thiolate bonding, together with lower binding-energy components associated with sulfide-containing species.^53^ The C 1s spectrum was deconvoluted into components corresponding to C–C, C–N, C=O/N–C=O, and carboxyl groups. The N 1s spectrum showed contributions from C–N and amino functionalities.^54^

**Figure 9:**
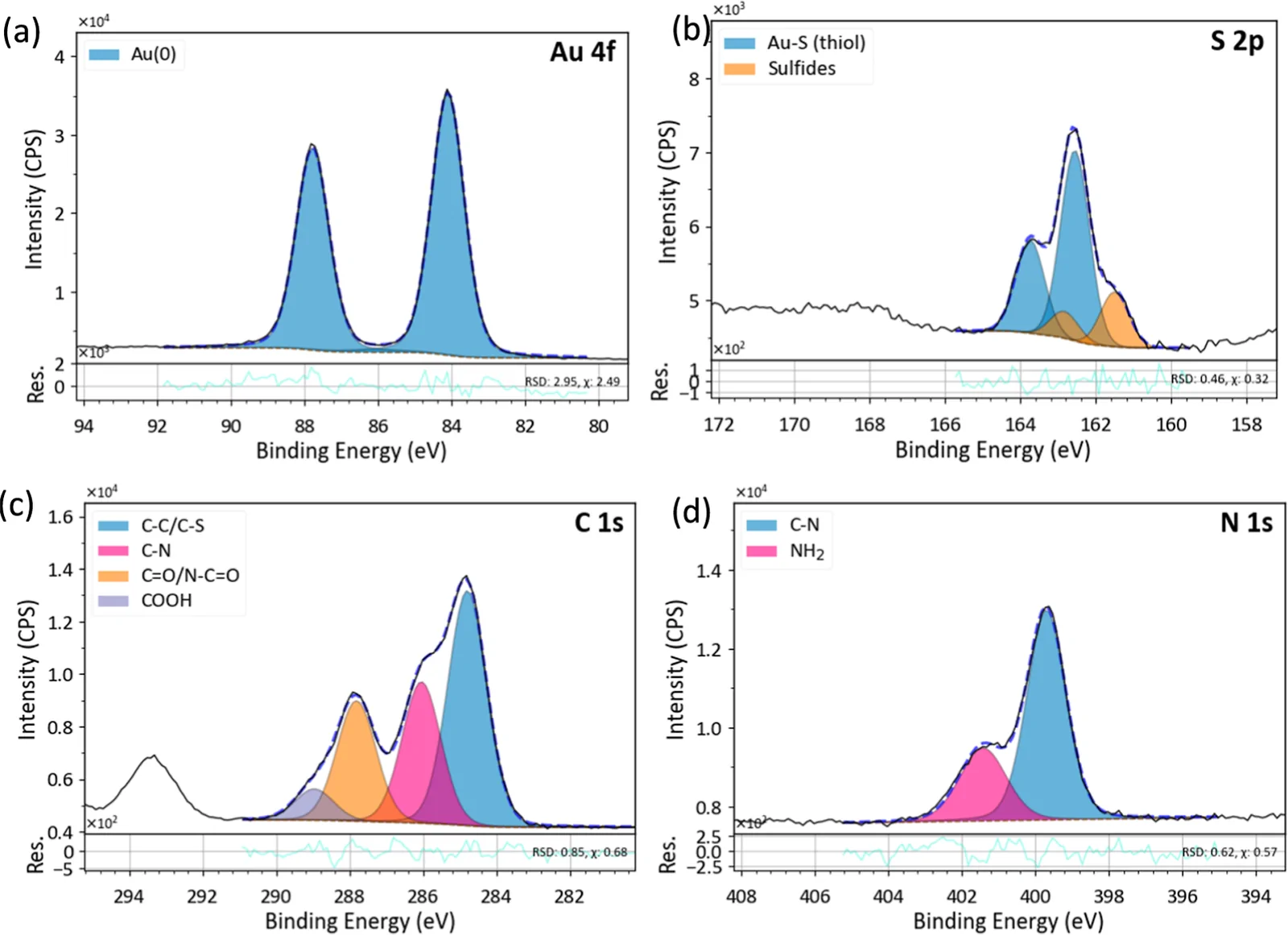
XPS spectra for NIR-emissive GSH-AuNCs: (a) Au 4f; (b) S 2p; (c) C 1s; and (d) N 1s.

Quantitative XPS analysis revealed increasing Au/S ratios of 1.64, 1.76, and 2.03 for red-, dual-, and NIR-emissive GSH-AuNCs, respectively. The trend was consistent with the SAXS results, where Guinier analysis yielded radii of 7.39 ± 0.27 Å, 9.2 ± 0.4 Å, and 9.8 ± 0.45 Å for the red-, dual-, and NIR-emissive samples, respectively. The increase in the Au/S ratio and cluster size with increasing precursor concentration is consistent with progressive growth of the gold component. Together with the CD evidence for changes in the Au­(I)-thiolate shell, these results indicate that both core nuclearity and ligand-shell organization evolve during synthesis. The resulting modification of the nanocluster electronic structure provides a plausible explanation for the observed red shift in fluorescence emission with increasing precursor concentration.^43,50,55^

Together, the SAXS, XPS, CD, and electrostatic measurements demonstrate that increasing the gold precursor concentration drives coupled but not identical changes in core size, metal–ligand coordination, and surface organization. The dual-emissive GSH-AuNCs therefore represent a structurally distinct intermediate state, characterized by intermediate core growth and a uniquely reorganized ligand shell, whereas further precursor increase promotes larger nuclearity clusters with enhanced Au incorporation and stronger surface-charge screening.

### 3.5 Targeted Detection of EpCAM-Expressing Cancer Cells Using Red- and NIR-Emissive GSH–AuNC–SYL3C–Aptamer Nanoprobes

Figure 10 presents the flow cytometry histogram of median fluorescent intensity (MFI) for EpCAM-positive PC3 and EpCAM-negative HEK 293 incubated with the red- or NIR-emissive GSH-AuNC-SYL3C-Aptamer nanoprobes. Fluorescence was detected using the BL3 (488 nm excitation, 695 ± 40 nm emission) and BL4 (488 nm excitation, 780 ± 60 nm emission) channels for the red and NIR-emissive probes, respectively. For both nanoprobes, PC3 cells exhibited higher MFI values than HEK293 cells, demonstrating selective binding of the SYL3C-functionalized GSH-AuNCs to EpCAM-expressing cells.^56–58^ Notably, although the absolute MFI recorded in the BL4 channel was lower than that measured in the BL3 channel, the NIR-emissive nanoprobe clearly discriminated between PC3 and HEK293 cell populations, confirming its effective targeting capability. The lower fluorescence intensity in the BL4 channel is likely attributable, at least in part, to the reduced detection efficiency of the flow cytometer at longer wavelengths. Nevertheless, fluorescence detection in the NIR region offers the intrinsic advantage of reduced biological autofluorescence, which is expected to improve signal-to-background contrast in biological imaging applications.^59–61^ The successful discrimination of EpCAM-positive from EpCAM-negative cells therefore demonstrates the potential of the NIR-emissive GSH-AuNCs–SYL3C nanoprobe for targeted cancer cell detection and provides a foundation for future NIR fluorescence imaging in more complex biological systems.

**Figure 10:**
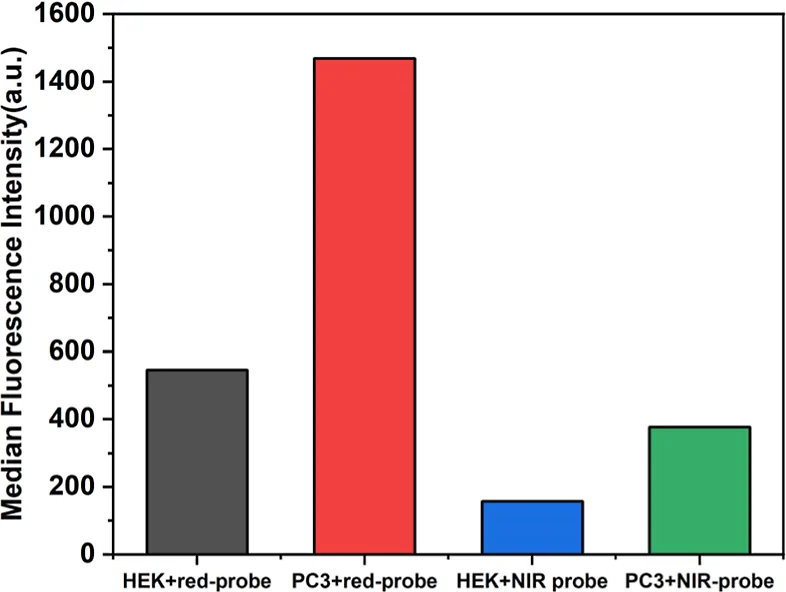
Median fluorescence intensity from flow cytometry for the HEK + probe and PC3 + probe in the BL3 (for the red probe) or BL4 (for the NIR probe) channel.

## 4 Conclusion

In this work, we systematically investigated the influence of synthesis conditions on the formation and photophysical properties of NIR-emissive GSH-AuNCs. The generation of NIR emission was found to depend sensitively on the combined effects of gold precursor concentration, reaction pH, and temperature, highlighting the narrow synthetic window required for optimal cluster formation. Under the optimized conditions (95 °C, 26 mM HAuCl_4_, and 80 μL of 1 M NaOH), highly fluorescent GSH-AuNCs exhibiting a single emission maximum at 825 nm were obtained, with a fluorescence intensity approximately 7.2 times higher than that of the conventionally synthesized 640 nm-emissive GSH-AuNCs after absorption correction.

Comprehensive spectroscopic and structural characterization revealed that tuning the gold precursor concentration modifies both the gold core and the Au­(I) thiolate ligand shell. Time-resolved fluorescence measurements demonstrated changes in excited-state dynamics accompanying the evolution from red- to NIR-emissive clusters, while circular dichroism spectroscopy revealed systematic reorganization of the ligand–metal coordination environment and Au­(I)-thiolate shell electronic structure. Zeta potential and density measurements further showed that changes in surface electrostatics and structural compactness do not evolve synchronously, indicating that metal core growth and ligand shell reorganization are only partially coupled. XPS confirmed similar chemical compositions and oxidation states across the differently emissive GSH-AuNCs, whereas the increasing Au/S ratio and larger particle sizes determined by SAXS demonstrated progressive growth of the gold core with increasing precursor concentration. Collectively, these results establish that the emission tuning of GSH-AuNCs arises from the combined evolution of core nuclearity, ligand-shell organization, and interfacial electrostatics rather than from a single structural parameter.

To demonstrate their potential for biomedical applications, both red- and NIR-emissive GSH-AuNCs were successfully functionalized with the EpCAM-targeting SYL3C aptamer. Flow cytometry confirmed selective recognition of EpCAM-expressing PC3 cancer cells over EpCAM-negative HEK cells for both nanoprobes, demonstrating that the NIR-emissive GSH-AuNCs retain their targeting capability while extending fluorescence detection into the NIR region. Overall, this work establishes a rational framework linking synthetic conditions with the structural evolution, electronic properties, and optical behavior of GSH-AuNCs, providing design principles for engineering gold nanoclusters with tailored photophysical and interfacial properties for biomedical imaging, biosensing, and imaging-guided therapeutic applications.

## Supplementary Material



## Notes

Figures S1–15: UV–vis absorption and emission spectra of GSH-AuNCs synthesized under different pH, concentration of gold, or temperature, including corresponding absorbance comparison at 265 nm and fluorescence intensity comparison of the peaks at 640 and 825 nm; Figures S16–17 TCSPC fluorescence lifetime decays for different emissive GSH-AuNCs; and Figure S18 SAXS-analyzed data of GSH-AuNCs with different emissions (PDF)

## References

[1] Liu B., Yang Z., Zhou J., Chen B., Li W., Hou J., Wang X., Ning S., Xu G., Zeng X. (2025). Gold nanoclusters as multifunctional nanoantibiotics: synthesis,
mechanisms and applications in combating bacterial infections. Chem. Eng. J..

[2] Mehdi J., Ansari V. A., Singh A. (2026). A Review of
Gold Nanoparticles: Synthesis
Methods and Biomedical Applications. Biomed.
Mater. & Devices.

[3] Genji
Srinivasulu Y., Yao Q., Goswami N., Xie J. (2020). Interfacial
engineering of gold nanoclusters for biomedical applications. Mater. Horiz..

[4] Russell B., Jachimska B., Kralka I., Mulheran P., Chen Y. (2016). Human serum
albumin encapsulated gold nanoclusters: effects of cluster synthesis
on natural protein characteristics. J. Mater.
Chem. B.

[5] Li H., Li H., Wan A. (2020). Luminescent
gold nanoclusters for in vivo tumor imaging. Analyst.

[6] Niazi S., Khan I. M., Akhtar W., ul Haq F., Pasha I., Khan M. K. I., Mohsin A., Ahmad S., Zhang Y., Wang Z. (2024). Aptamer functionalized
gold nanoclusters as an emerging nanoprobe
in biosensing, diagnostic, catalysis and bioimaging. Talanta.

[7] Jiang X., Du B., Huang Y., Yu M., Zheng J. (2020). Cancer photothermal
therapy with ICG-conjugated gold nanoclusters. Bioconjugate Chem..

[8] Alkudaisi N., Russell B. A., Birch D. J., Chen Y. (2019). Lysozyme encapsulated
gold nanoclusters for probing the early stage of lysozyme aggregation
under acidic conditions. J. Photochem. Photobiol.,
B.

[9] Russell B., Mulheran P., Birch D., Chen Y. (2016). Probing the Sudlow
binding site with warfarin: how does gold nanocluster growth alter
human serum albumin?. Phys. Chem. Chem. Phys..

[10] Alkudaisi N., Russell B. A., Jachimska B., Birch D. J., Chen Y. (2019). Detecting
lysozyme unfolding via the fluorescence of lysozyme encapsulated gold
nanoclusters. J. Mater. Chem. B.

[11] Baghdasaryan A., Dai H. (2025). Molecular Gold Nanoclusters
for Advanced NIR-II Bioimaging and Therapy. Chem. Rev..

[12] Xu M., Qi Y., Liu G., Song Y., Jiang X., Du B. (2023). Size-dependent
in vivo transport of nanoparticles: implications for delivery, targeting,
and clearance. ACS Nano.

[13] Russell B., Kubiak-Ossowska K., Mulheran P., Birch D., Chen Y. (2015). Locating the
nucleation sites for protein encapsulated gold nanoclusters: a molecular
dynamics and fluorescence study. Phys. Chem.
Chem. Phys..

[14] Quek C.-H., Leong K. W. (2012). Near-infrared fluorescent
nanoprobes for in vivo optical
imaging. Nanomaterials.

[15] Chang X.-H., Zhang J., Wu L.-H., Peng Y.-K., Yang X.-Y., Li X.-L., Ma A.-J., Ma J.-C., Chen G.-Q. (2019). Research
progress of near-infrared fluorescence immunoassay. Micromachines.

[16] Zhang F., Tang B. Z. (2021). Near-infrared luminescent
probes for bioimaging and
biosensing. Chem. Sci..

[17] Sun Y., Wu J., Wang C., Zhao Y., Lin Q. (2017). Tunable near-infrared
fluorescent gold nanoclusters: Temperature sensor and targeted bioimaging. New J. Chem..

[18] Tu X., Chen W., Guo X. (2011). Facile one-pot synthesis of near-infrared
luminescent gold nanoparticles for sensing copper (II). Nanotechnology.

[19] Chen L.-Y., Wang C.-W., Yuan Z., Chang H.-T. (2015). Fluorescent gold
nanoclusters: recent advances in sensing and imaging. Anal. Chem..

[20] Shichibu Y., Negishi Y., Tsukuda T., Teranishi T. (2005). Large-scale
synthesis of thiolated Au25 clusters via ligand exchange reactions
of phosphine-stabilized Au11 clusters. J. Am.
Chem. Soc..

[21] Madhu M., Tseng W.-B., Chou Y.-S., Krishna Kumar A. S., Lu C.-Y., Chang P.-L., Tseng W.-L. (2024). Peptide-Directed
Synthesis of Aggregation-Induced Emission Enhancement-Active Gold
Nanoclusters for Single-and Two-Photon Imaging of Lysosome and Expressed
αvβ3 Integrin Receptors. Anal. Chem..

[22] Jin K., Wang W., Qi G., Peng X., Gao H., Zhu H., He X., Zou H., Yang L., Yuan J. (2023). An explainable machine-learning
approach for revealing the complex
synthesis path–property relationships of nanomaterials. Nanoscale.

[23] Mukherjee M., Chatterjee A., Purkayastha P. (2025). Size Dependent Liposomal Fusion and
H-Bonded Sticky Aggregation Induced by Gold Nanoclusters. J. Phys. Chem. B.

[24] Wang E., Xu W. W., Zhu B., Gao Y. (2021). Understanding
the Chemical
Insights of Staple Motifs of Thiolate-Protected Gold Nanoclusters. Small.

[25] Sharma N., Mohammad W., Le Guével X., Shanavas A. (2024). Gold nanoclusters as
high resolution NIR-II theranostic agents. Chem.
Biomed. Imaging.

[26] Pang Z., Yan W., Yang J., Li Q., Guo Y., Zhou D., Jiang X. (2022). Multifunctional gold nanoclusters for effective targeting, near-infrared
fluorescence imaging, diagnosis, and treatment of cancer lymphatic
metastasis. ACS Nano.

[27] Wang N., Liu H., Hao J., Bai X., Li H., Zhang Z., Wang H., Tang J. (2015). Single molecular recognition
force
spectroscopy study of a DNA aptamer with the target epithelial cell
adhesion molecule. Analyst.

[28] Song Y., Zhu Z., An Y., Zhang W., Zhang H., Liu D., Yu C., Duan W., Yang C. J. (2013). Selection of DNA aptamers against
epithelial cell adhesion molecule for cancer cell imaging and circulating
tumor cell capture. Anal. Chem..

[29] Kim M., Kim D.-M., Kim K.-S., Jung W., Kim D.-E. (2018). Applications
of cancer cell-specific aptamers in targeted delivery of anticancer
therapeutic agents. Molecules.

[30] Amaya-González S., De-los-Santos-Álvarez N., Miranda-Ordieres A. J., Lobo-Castañón M. J. (2013). Aptamer-based
analysis: A promising
alternative for food safety control. Sensors.

[31] Wang Y., Li J., Qiao P., Jing L., Song Y., Zhang J., Chen Q., Han Q. (2018). Screening and application of a new
aptamer for the rapid detection of Sudan dye III. Eur. J. Lipid Sci. Technol..

[32] Wu D., Gordon C. K., Shin J. H., Eisenstein M., Soh H. T. (2022). Directed evolution
of aptamer discovery technologies. Acc. Chem.
Res..

[33] Wang Z., Zhang Y., Wu L., Chen J., Xie S., He J., Zhang Q., Chen H., Chen F., Liu Y. (2023). An Aptamer-Functionalized
DNA Circuit to Establish an Artificial
Interaction between T Cells and Cancer Cells. Angew. Chem., Int. Ed..

[34] Kolovskaya O. S., Zyuzyukina A. V., Dassie J. P., Zamay G. S., Zamay T. N., Boyakova N. V., Khorzhevskii V. A., Kirichenko D. A., Lapin I. N., Shchugoreva I. A. (2023). Monitoring of breast
cancer progression via aptamer-based detection of circulating tumor
cells in clinical blood samples. Front. Mol.
Biosci..

[35] Wu D., Feagin T., Mage P., Rangel A., Wan L., Kong D., Li A., Coller J., Eisenstein M., Soh H. (2023). Flow-cell-based technology
for massively parallel characterization
of base-modified DNA aptamers. Anal. Chem..

[36] Kerherve G., Skinner W. S. J., Hochhaus J. A., Graf A., Morgan D. J., Isaacs M. A., Reed B. P., Nakajima H., Payne D. J. (2026). KherveFitting:
An Open Source Software for Fitting X-Ray Photoelectron Spectroscopy
Data. Surf. Interface Anal..

[37] Doveiko D., Martin A. R. G., Vyshemirsky V., Stebbing S., Kubiak-Ossowska K., Rolinski O., Birch D. J. S., Chen Y. (2024). Nanoparticle Metrology
of Silicates Using Time-Resolved Multiplexed Dye Fluorescence Anisotropy,
Small Angle X-ray Scattering, and Molecular Dynamics Simulations. Materials.

[38] Hopkins J. B., Gillilan R. E., Skou S. (2017). BioXTAS RAW:
improvements to a free
open-source program for small-angle X-ray scattering data reduction
and analysis. J. Appl. Crystallogr..

[39] Luo Z., Yuan X., Yu Y., Zhang Q., Leong D. T., Lee J. Y., Xie J. (2012). From aggregation-induced emission
of Au (I)–thiolate complexes to ultrabright Au (0)@ Au (I)–thiolate
core–shell nanoclusters. J. Am. Chem.
Soc..

[40] Mbalaha Z.
S., Edwards P. R., Birch D. J. S., Chen Y. (2019). Synthesis of Small
Gold Nanorods and Their Subsequent Functionalization with Hairpin
Single Stranded DNA. ACS Omega.

[41] Wei G., Yu J., Wang J., Gu P., Birch D. J., Chen Y. (2016). Hairpin DNA-functionalized
gold nanorods for mRNA detection in homogenous solution. J. Biomed. Opt..

[42] Mbalaha Z., Craig G., Birch D., Chen Y. (2024). Aptamer functionalised
small gold nanorod-based nanoprobes: targeting EpCAM over-expressed
cancer cells for photothermal therapy. Austin
J. Anal. Pharm. Chem..

[43] Negishi Y., Nobusada K., Tsukuda T. (2005). Glutathione-protected gold clusters
revisited: bridging the gap between gold (I)– thiolate complexes
and thiolate-protected gold nanocrystals. J.
Am. Chem. Soc..

[44] Bai X., Xu S., Wang L. (2018). Full-range pH stable Au-clusters in nanogel for confinement-enhanced
emission and improved sulfide sensing in living cells. Anal. Chem..

[45] Jin R. (2015). Atomically
precise metal nanoclusters: stable sizes and optical properties. Nanoscale.

[46] Häkkinen H. (2008). Atomic and
electronic structure of gold clusters: understanding flakes, cages
and superatoms from simple concepts. Chem. Soc.
Rev..

[47] Pramanik G., Humpolickova J., Valenta J., Kundu P., Bals S., Bour P., Dracinsky M., Cigler P. (2018). Gold nanoclusters with
bright near-infrared photoluminescence. Nanoscale.

[48] Cheng D., Liu R., Hu K. (2022). Gold nanoclusters:
Photophysical properties and photocatalytic
applications. Front. Chem..

[49] Zhou M., Zeng C., Li Q., Higaki T., Jin R. (2019). Gold nanoclusters:
bridging gold complexes and plasmonic nanoparticles in photophysical
properties. Nanomaterials.

[50] Stamplecoskie K. G., Kamat P. V. (2014). Size-dependent excited state behavior of glutathione-capped
gold clusters and their light-harvesting capacity. J. Am. Chem. Soc..

[51] Zhu F., Li X., Li Y., Yan M., Liu S. (2015). Enantioselective circular
dichroism sensing of cysteine and glutathione with gold nanorods. Anal. Chem..

[52] Gautier C., Bürgi T. (2008). Chiral inversion of gold nanoparticles. J. Am. Chem. Soc..

[53] Li Z., Liang S., Zhang C., Zhou L., Luo F., Lou Z., Chen Z., Zhang X., Yang M. (2024). A ratiometric fluorescence
and colorimetry dual-signal sensing strategy based on o-phenylenediamine
and AuNCs for determination of Cu2+ and glyphosate. Microchim. Acta.

[54] Kong L., Chu X., Ling X., Ma G., Yao Y., Meng Y., Liu W. (2016). Biocompatible glutathione-capped
gold nanoclusters for dual fluorescent
sensing and imaging of copper (II) and temperature in human cells
and bacterial cells. Microchim. Acta.

[55] Zhang G., Fang B., Peng J., Deng S., Hu L., Lai W. (2025). Luminescent gold nanoclusters
from synthesis to sensing: A comprehensive
review. Chem. Eng. J..

[56] Bohrmann L., Burghardt T., Haynes C., Saatchi K., Häfeli U. O. (2022). Aptamers
used for molecular imaging and theranostics-recent developments. Theranostics.

[57] Jenkins S. V., Nima Z. A., Vang K. B., Kannarpady G., Nedosekin D. A., Zharov V. P., Griffin R. J., Biris A. S., Dings R. P. (2017). Triple-negative breast cancer targeting
and killing
by EpCAM-directed, plasmonically active nanodrug systems. npj Precis. Oncol..

[58] Mitra M., Kandalam M., Rangasamy J., Shankar B., Maheswari U. K., Swaminathan S., Krishnakumar S. (2013). Novel epithelial cell adhesion molecule
antibody conjugated polyethyleneimine-capped gold nanoparticles for
enhanced and targeted small interfering RNA delivery to retinoblastoma
cells. Mol. Vision.

[59] Zhu S., Yung B. C., Chandra S., Niu G., Antaris A. L., Chen X. (2018). Near-infrared-II (NIR-II) bioimaging via off-peak NIR-I fluorescence
emission. Theranostics.

[60] Hong G., Antaris A. L., Dai H. (2017). Near-infrared fluorophores for biomedical
imaging. Nat. Biomed. Eng..

[61] Schmidt E. L., Ou Z., Ximendes E., Cui H., Keck C. H., Jaque D., Hong G. (2024). Near-infrared II fluorescence imaging. Nat.
Rev. Methods Primers.

