# Supplementary material for: Correlating Synthesis Conditions with Structural Evolution and Near-Infrared Emission in Glutathione-Capped Gold Nanoclusters
Source: ACS Omega. 2026 Oct 12;11(42):64501–12. doi: 10.1021/acsomega.6c03255 (PMC13717793; doi:10.1021/acsomega.6c03255)
Supplement: Supplementary file 1 [file ao6c03255_si_001.pdf]

Correlating Synthesis Conditions with Structural Evolution and Near-Infrared Emission in Glutathione-Capped Gold Nanoclusters

Bowen Wang[1], Nicolas Laurand[2], Barbara Jachimska[3], Sanjay Sandhu[4], Daniel Doveiko[1],

Amrutha Sankar[1], Christopher Kelly[5], Aruna Ivaturi[4], Yu Chen[1]*

[1] Photophysics Group, Centre for Molecular Nanometrology, Department of Physics, Scottish Universities Physics Alliance, University of Strathclyde, 107 Rottenrow, Glasgow G4 0NG, UK

[2] Institute of Photonics, Department of Physics, SUPA, University of Strathclyde, Glasgow G1 1RD, UK

[3]Jerzy Haber Institute of Catalysis and Surface Chemistry, Polish Academy of Sciences, Krakow, Poland.

[4] Smart Material Research and Device Technology (SMaRDT) Group, Department of Pure and Applied Chemistry, University of Strathclyde, Thomas Graham Building, 293 Cathedral Street, Glasgow, G1 1XL, UK

[5] School of Chemistry, University of Glasgow, University Avenue, Glasgow G12 8QQ, UK.

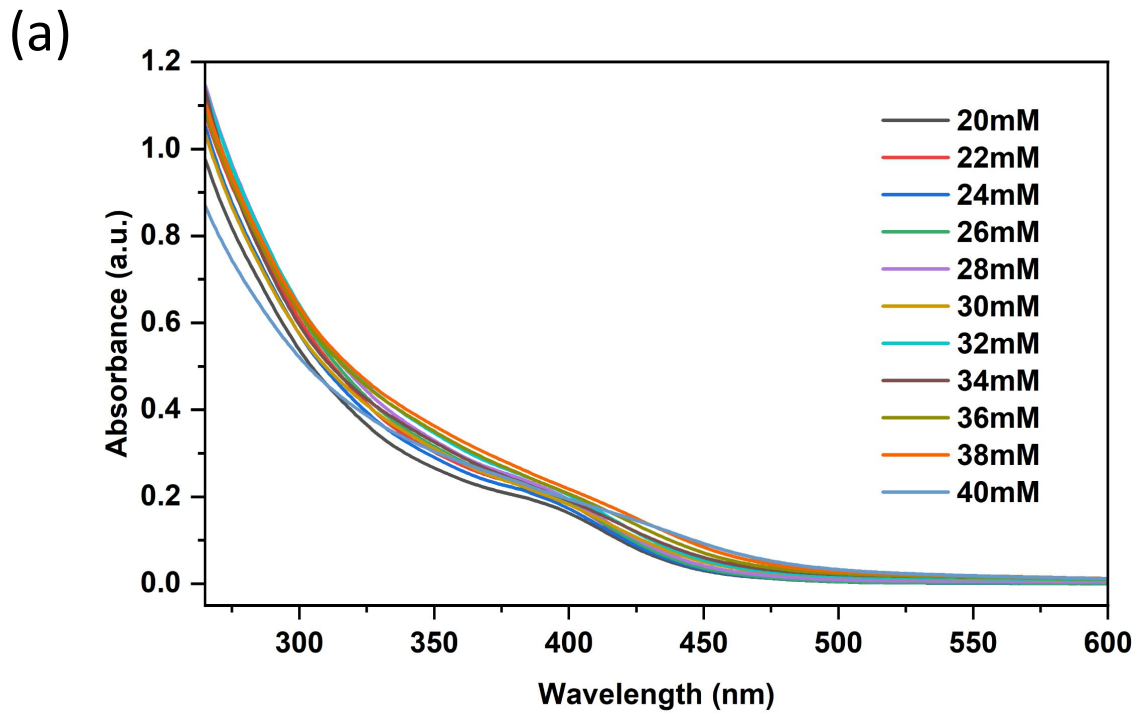

Figure S1. UV-Vis absorption spectra(a) and absorbance comparison at 265nm(b) for GSH-AuNCs synthesised at 70°C, original pH, different $C_{(Au)}$.

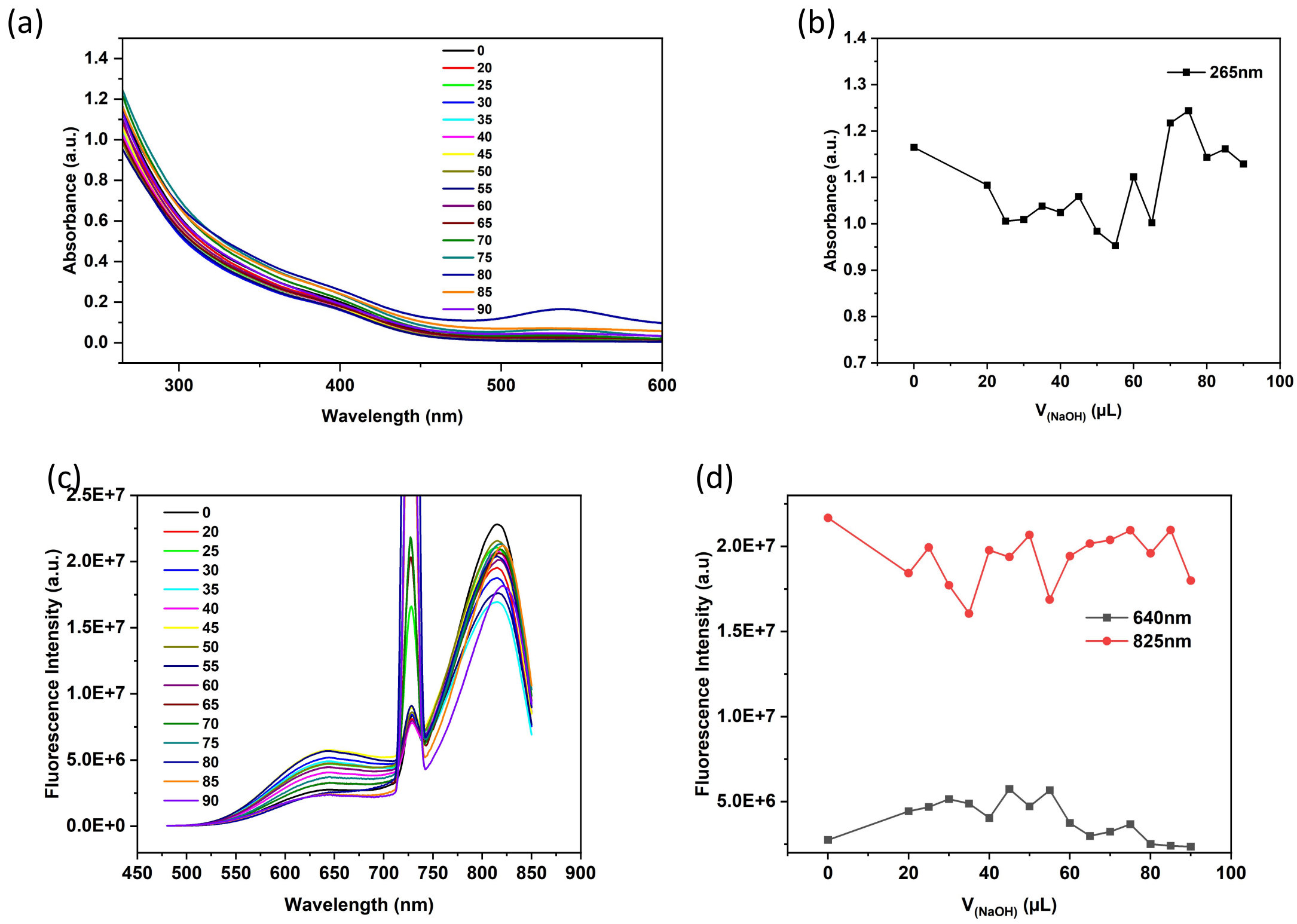

Figure S2. UV-Vis absorption spectra(a), absorbance comparison at 265nm(b), emission spectra(c) and intensity comparison at 640nm and 825nm(d) for GSH-AuNCs synthesised at 70°C, $C_{(Au)}$=34mM, different pH.

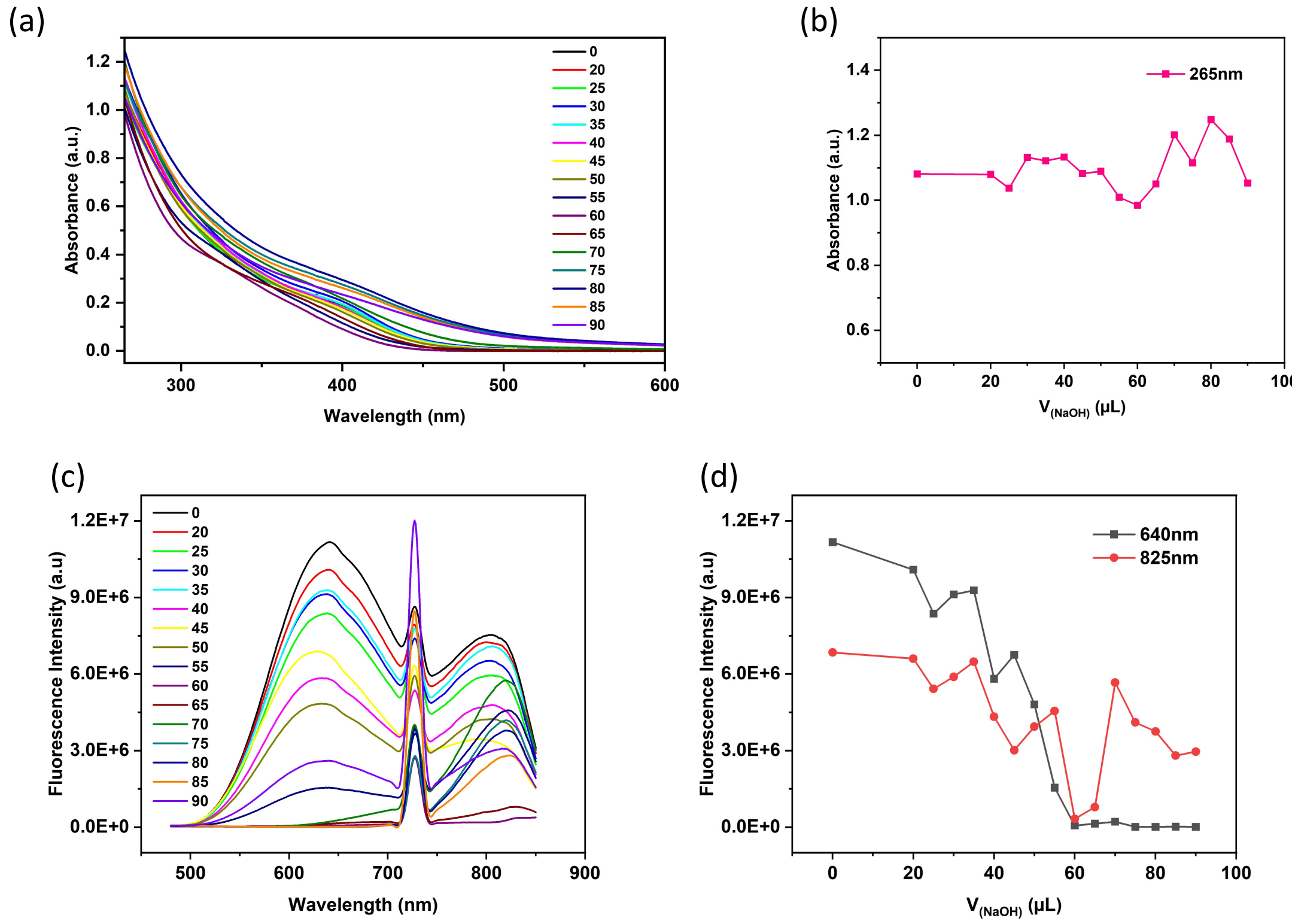

Figure S3. UV-Vis absorption spectra(a), absorbance comparison at 265nm(b), emission spectra(c) and intensity comparison at 640nm and 825nm(d) for GSH-AuNCs synthesised at 80°C, $C_{(Au)}$=20mM, different pH.

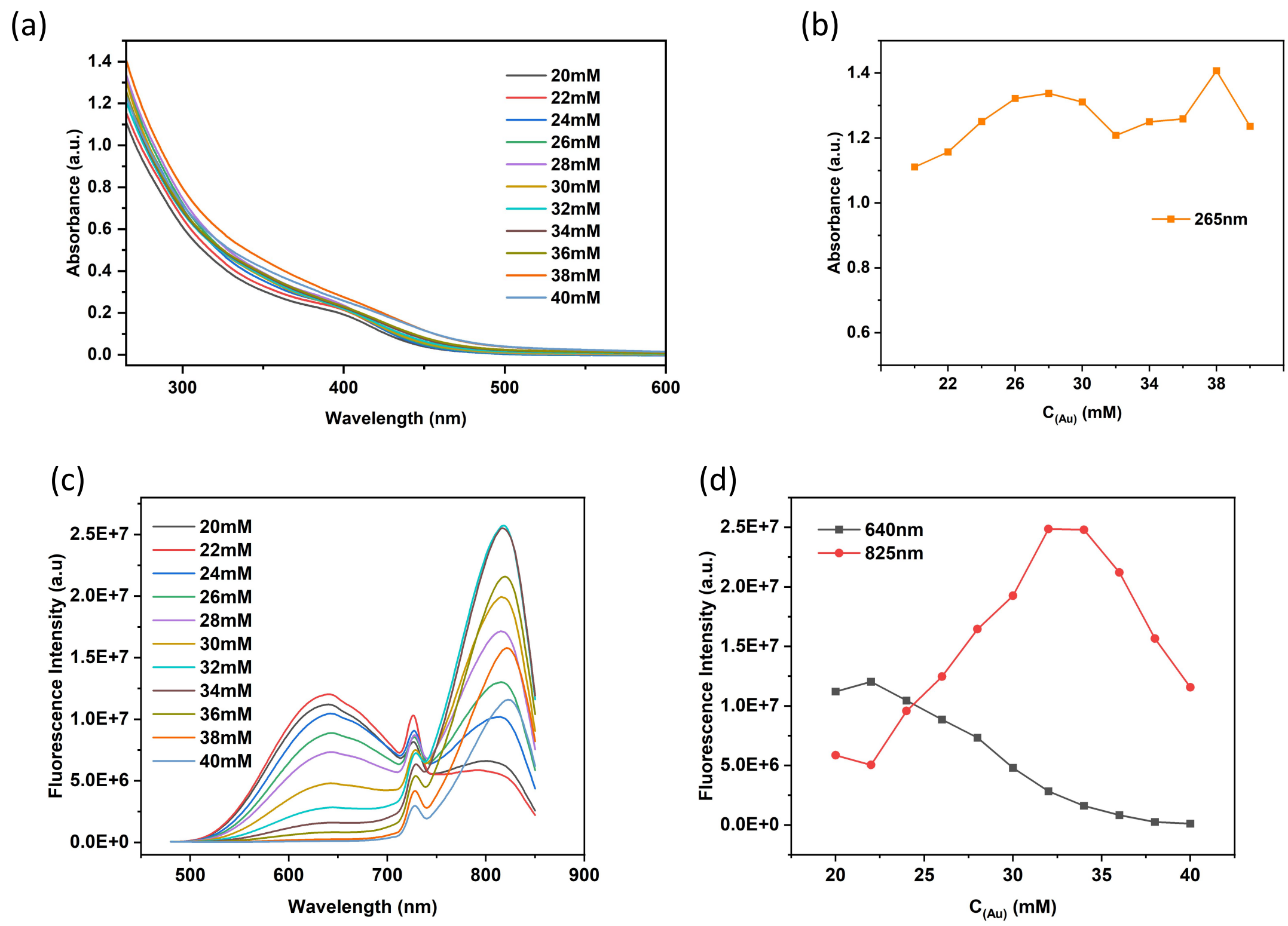

Figure S4. UV-Vis absorption spectra(a), absorbance comparison at 265nm(b), emission spectra(c) and intensity comparison at 640nm and 825nm(d) for GSH-AuNCs synthesised at 80°C, original pH, different $C_{(Au)}$.

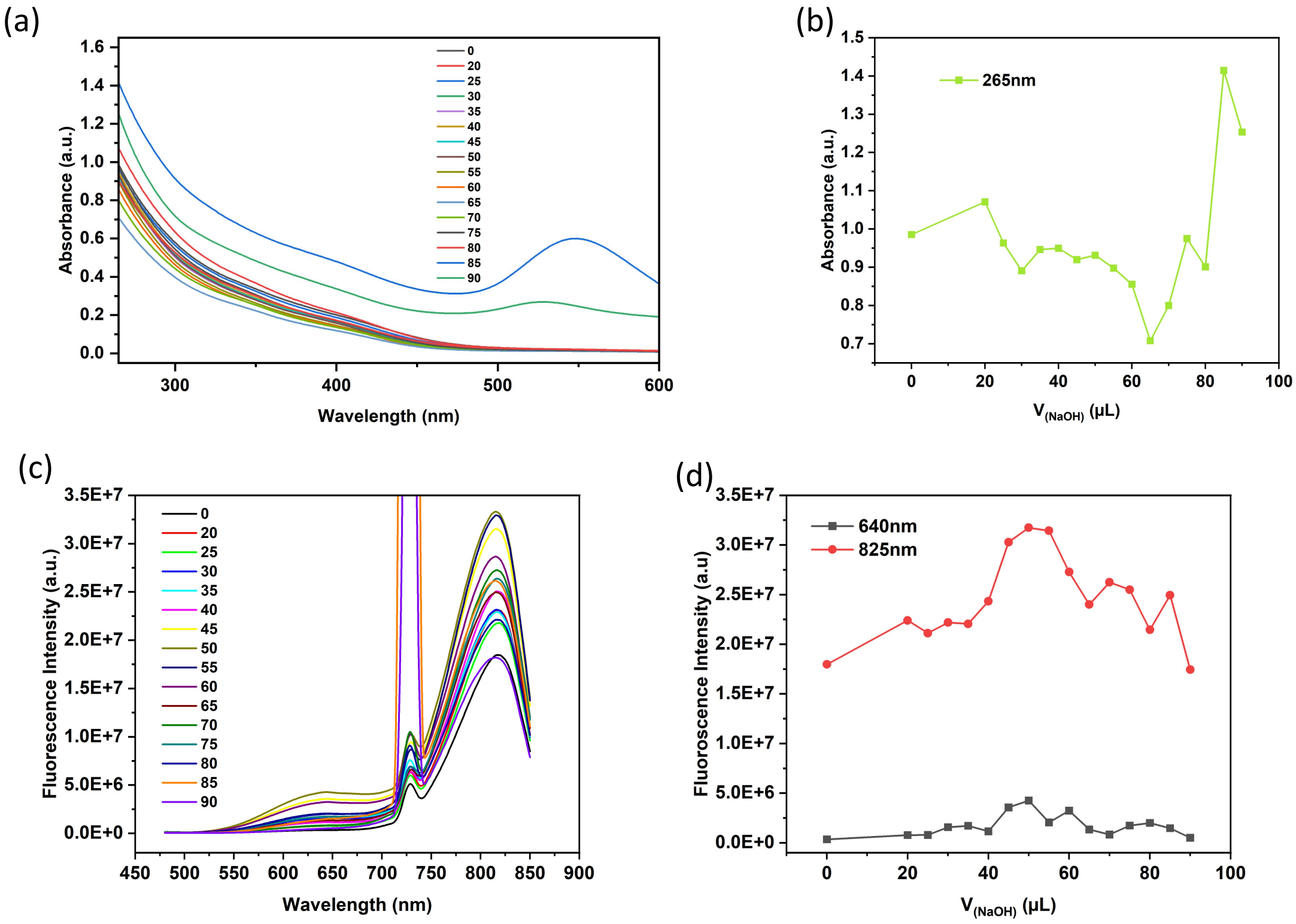

Figure S5. UV-Vis absorption spectra(a), absorbance comparison at 265nm(b), emission spectra(c) and intensity comparison at 640nm and 825nm(d) for GSH-AuNCs synthesised at 80°C, $C_{(Au)}$=34mM, different pH.

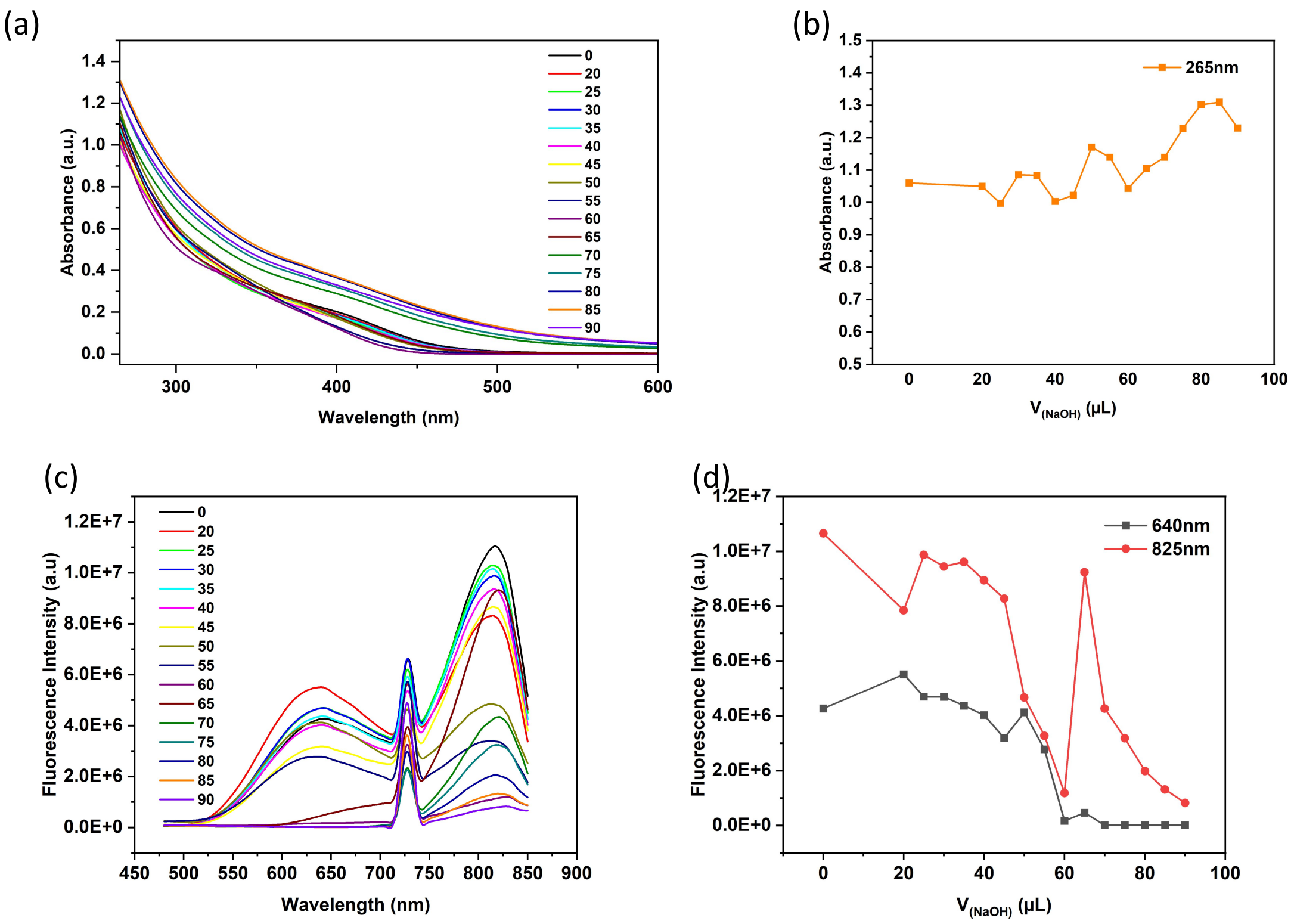

Figure S6. UV-Vis absorption spectra(a), absorbance comparison at 265nm(b), emission spectra(c) and intensity comparison at 640nm and 825nm(d) for GSH-AuNCs synthesised at 90°C, $C_{(Au)}$=20mM, different pH.

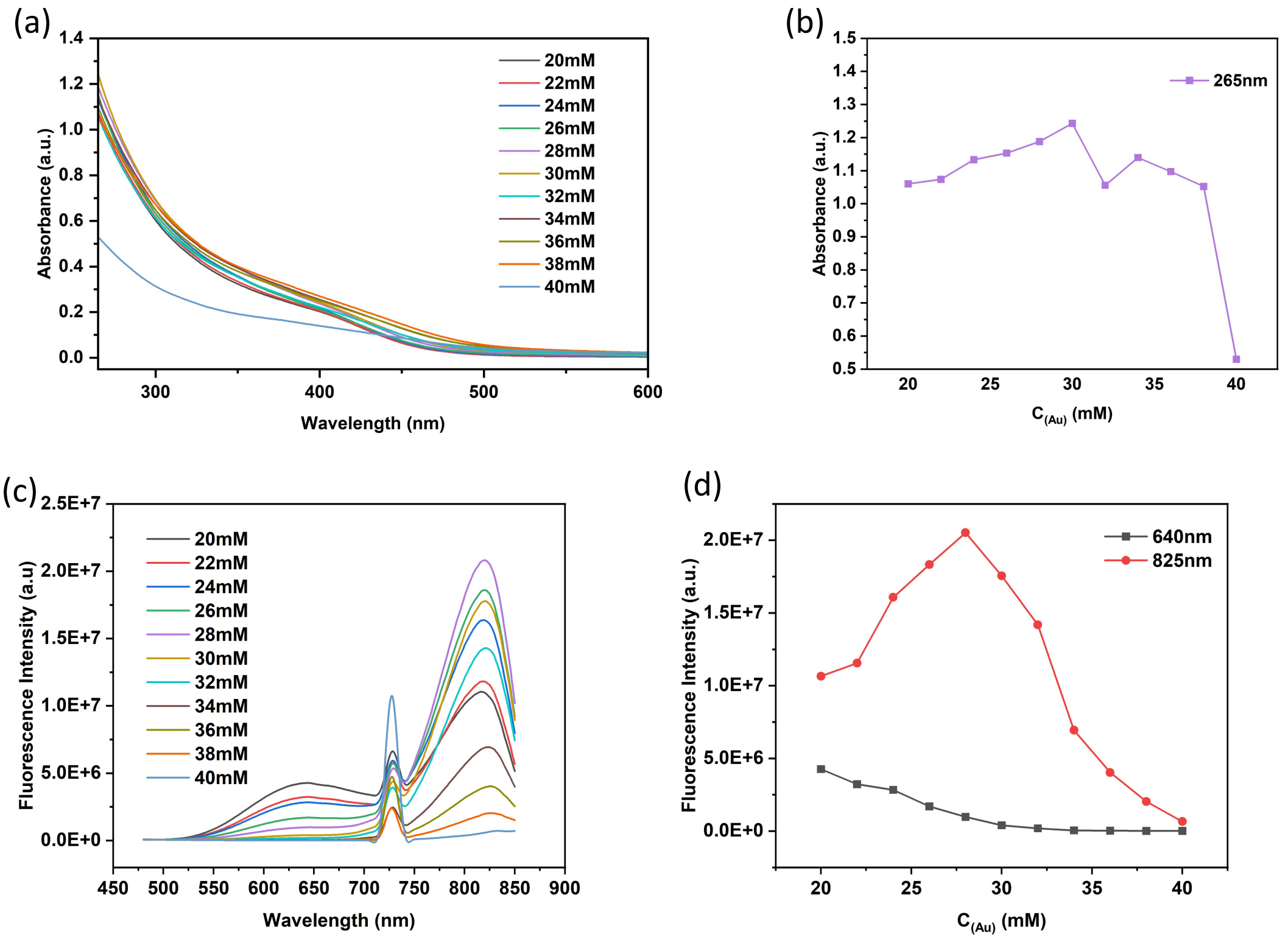

Figure S7. UV-Vis absorption spectra(a), absorbance comparison at 265nm(b), emission spectra(c) and intensity comparison at 640nm and 825nm(d) for GSH-AuNCs synthesised at 90°C, original pH, different $C_{(Au)}$.

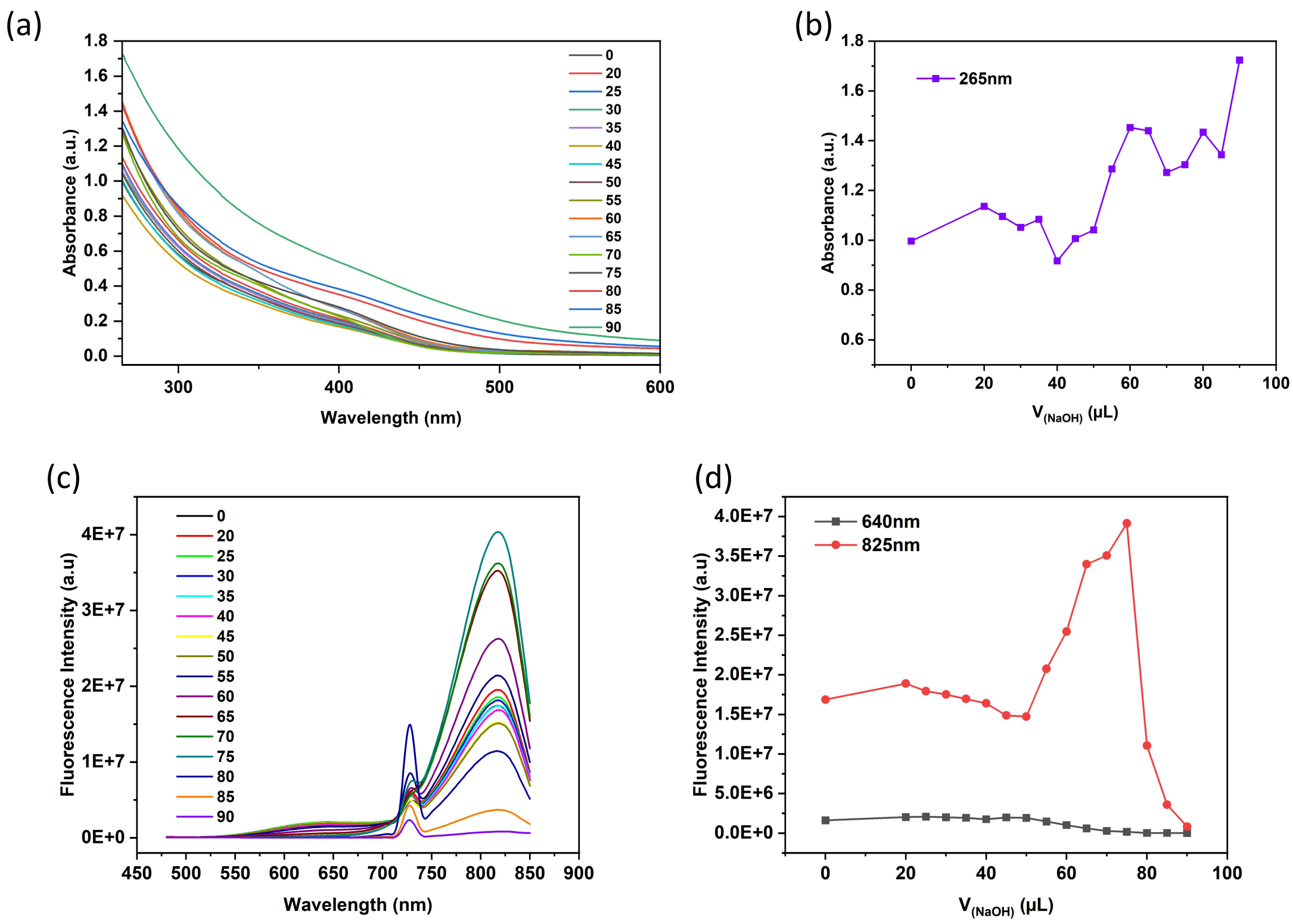

Figure S8. UV-Vis absorption spectra(a), absorbance comparison at 265nm(b), emission spectra(c) and intensity comparison at 640nm and 825nm(d) for GSH-AuNCs synthesised at 90°C, $C_{(Au)}$=26mM, different pH.

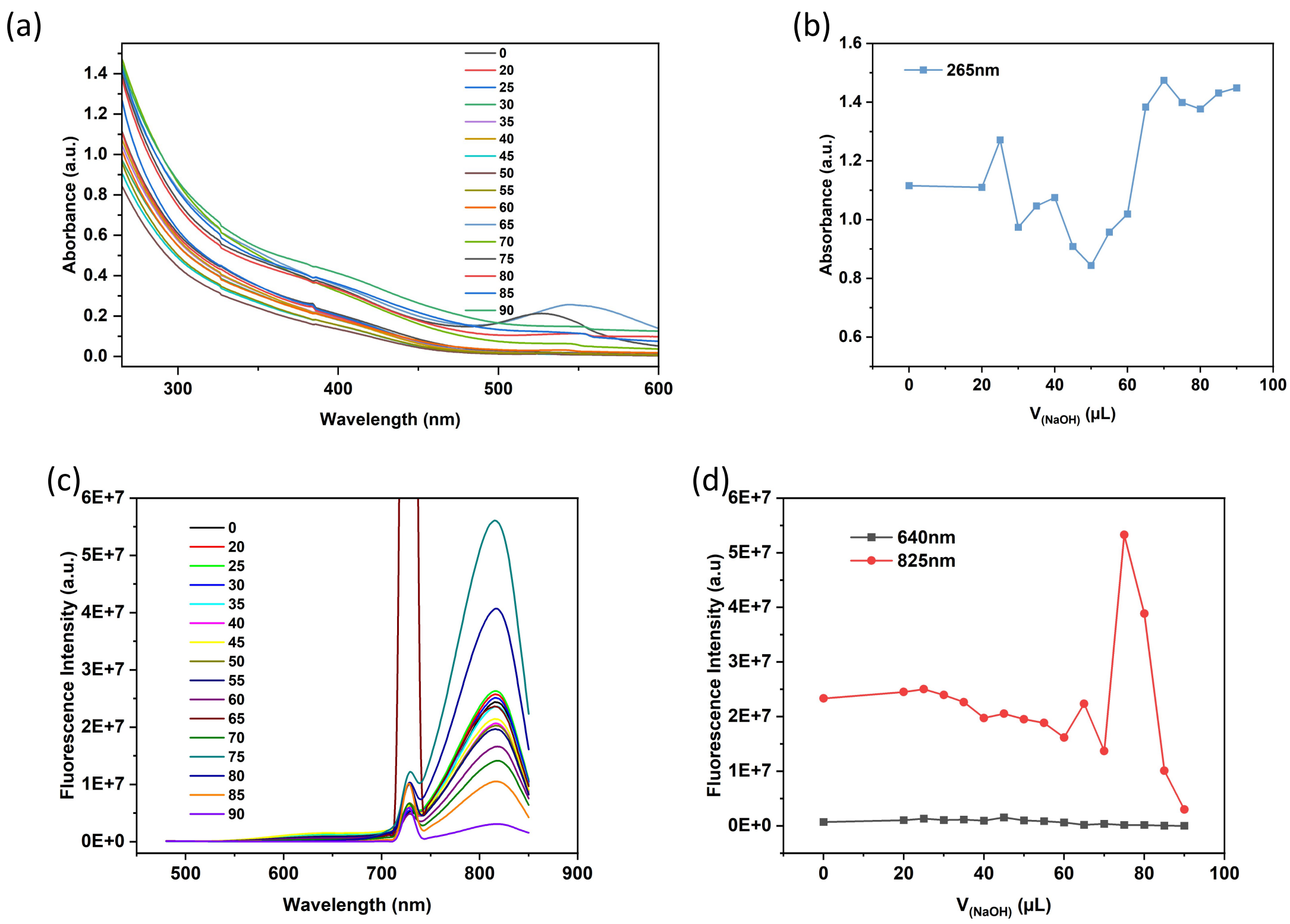

Figure S9. UV-Vis absorption spectra(a), absorbance comparison at 265nm(b), emission spectra(c) and intensity comparison at 640nm and 825nm(d) for GSH-AuNCs synthesised at 90°C, $C_{(Au)}$=28mM, different pH.

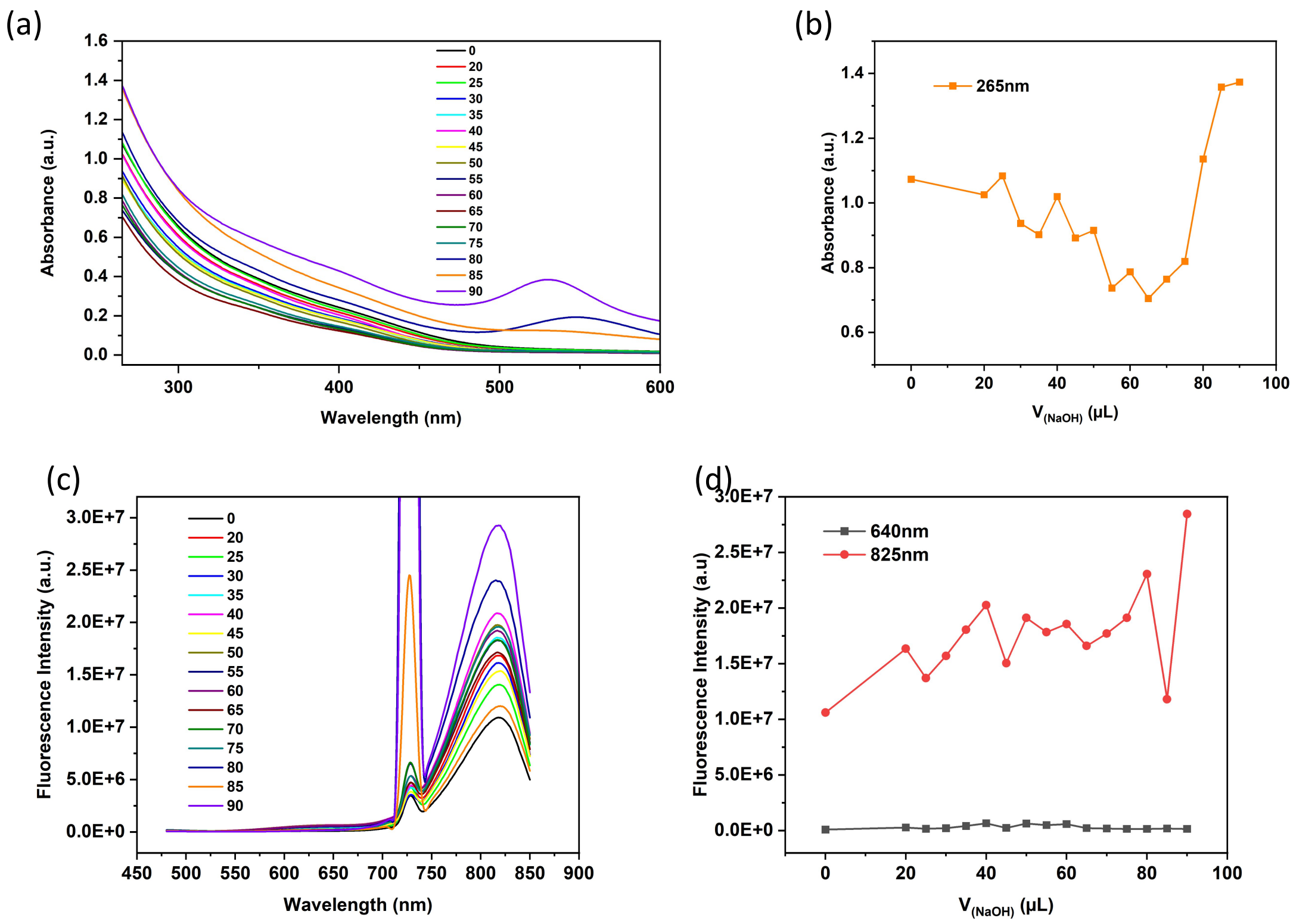

Figure S10. UV-Vis absorption spectra(a), absorbance comparison at 265nm(b), emission spectra(c) and intensity comparison at 640nm and 825nm(d) for GSH-AuNCs synthesised at 90°C, $C_{(Au)}$=34mM, different pH.

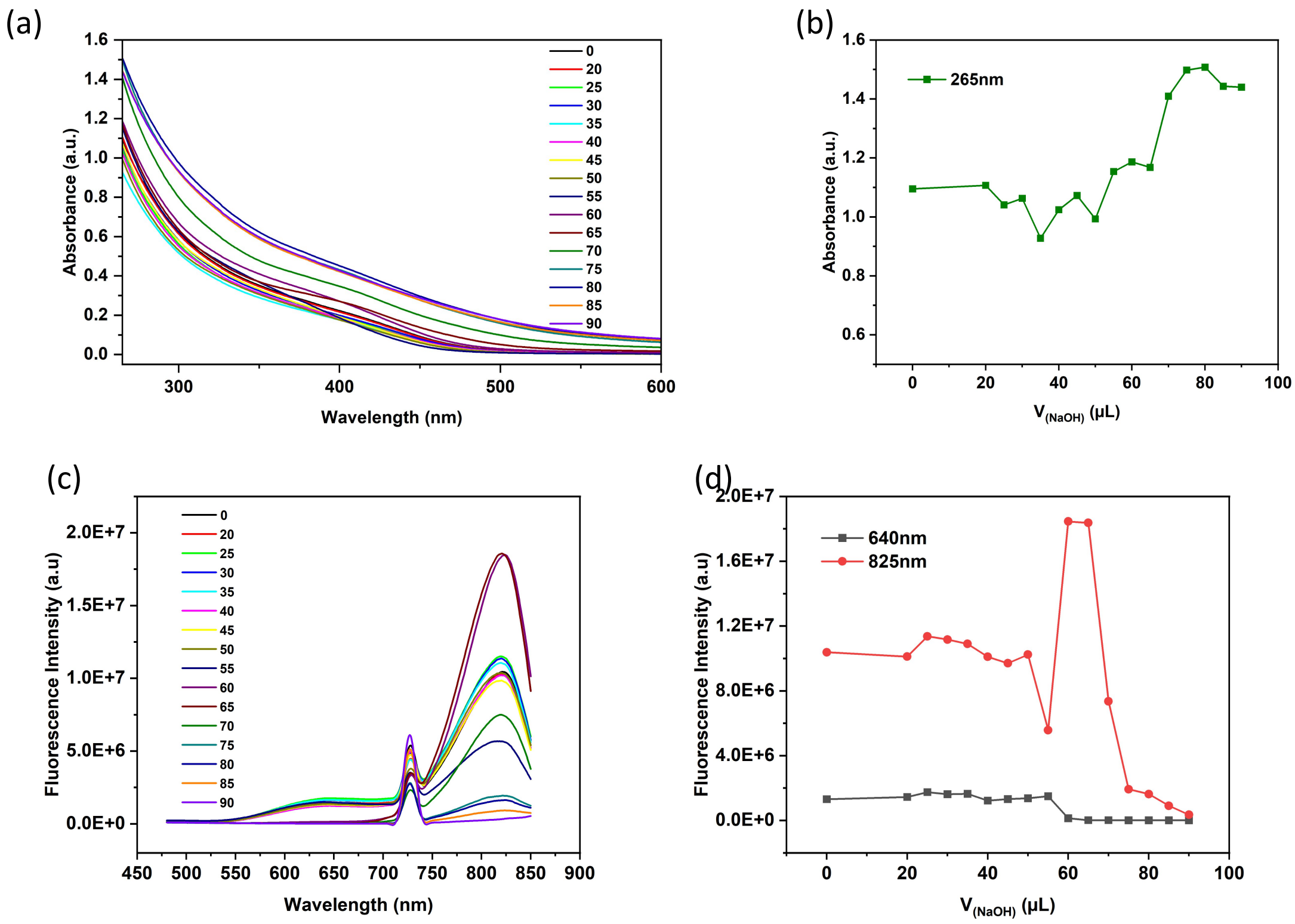

Figure S11. UV-Vis absorption spectra(a), absorbance comparison at 265nm(b), emission spectra(c) and intensity comparison at 640nm and 825nm(d) for GSH-AuNCs synthesised at 95°C, $C_{(Au)}$=20mM, different pH.

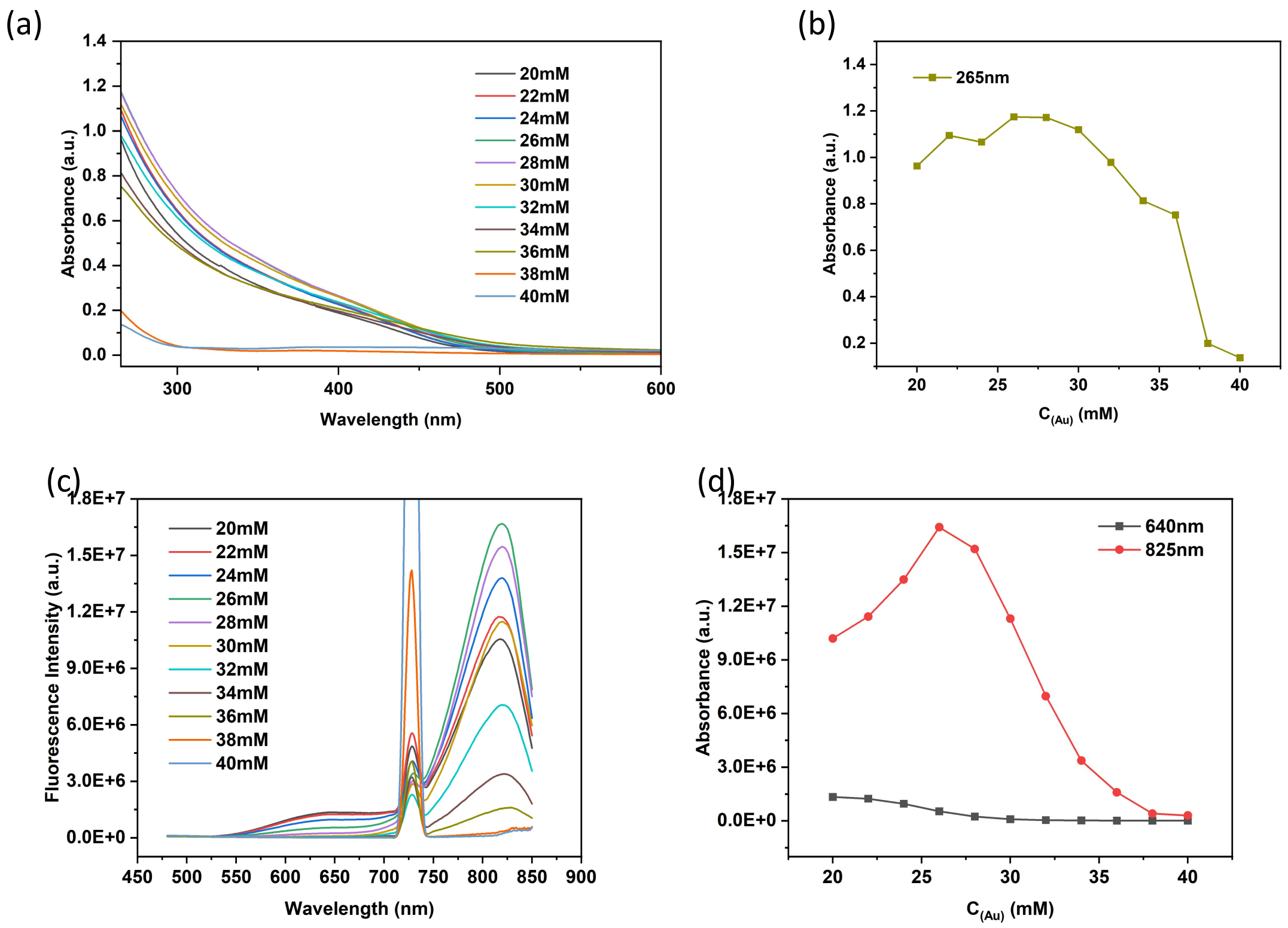

Figure S12. UV-Vis absorption spectra(a), absorbance comparison at 265nm(b), emission spectra(c) and intensity comparison at 640nm and 825nm(d) for GSH-AuNCs synthesised at 95°C, original pH, different $C_{(Au)}$.

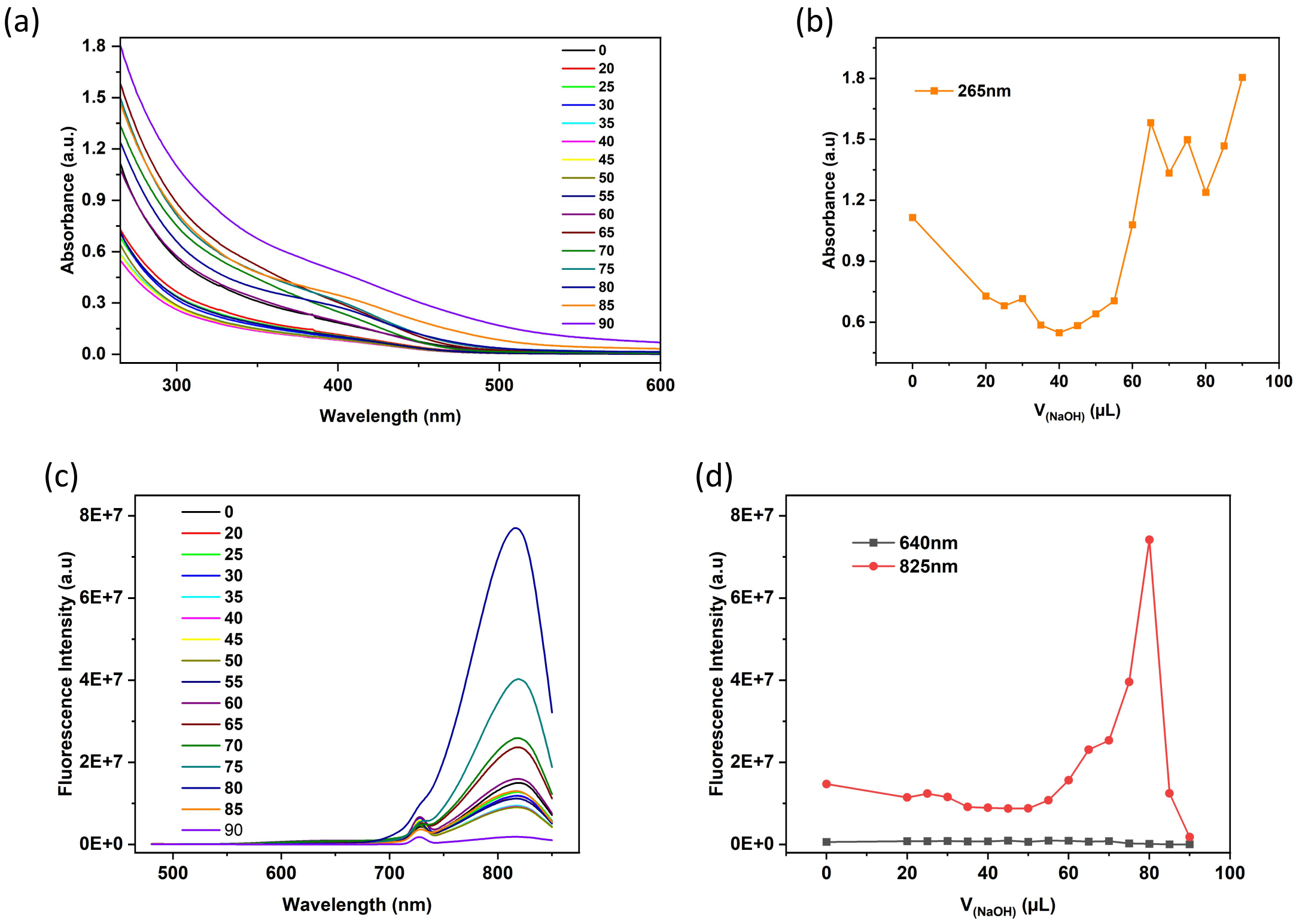

Figure S13. UV-Vis absorption spectra(a), absorbance comparison at 265nm(b), emission spectra(c) and intensity comparison at 640nm and 825nm(d) for GSH-AuNCs synthesised at 95°C, $C_{(Au)}$=26mM, different pH.

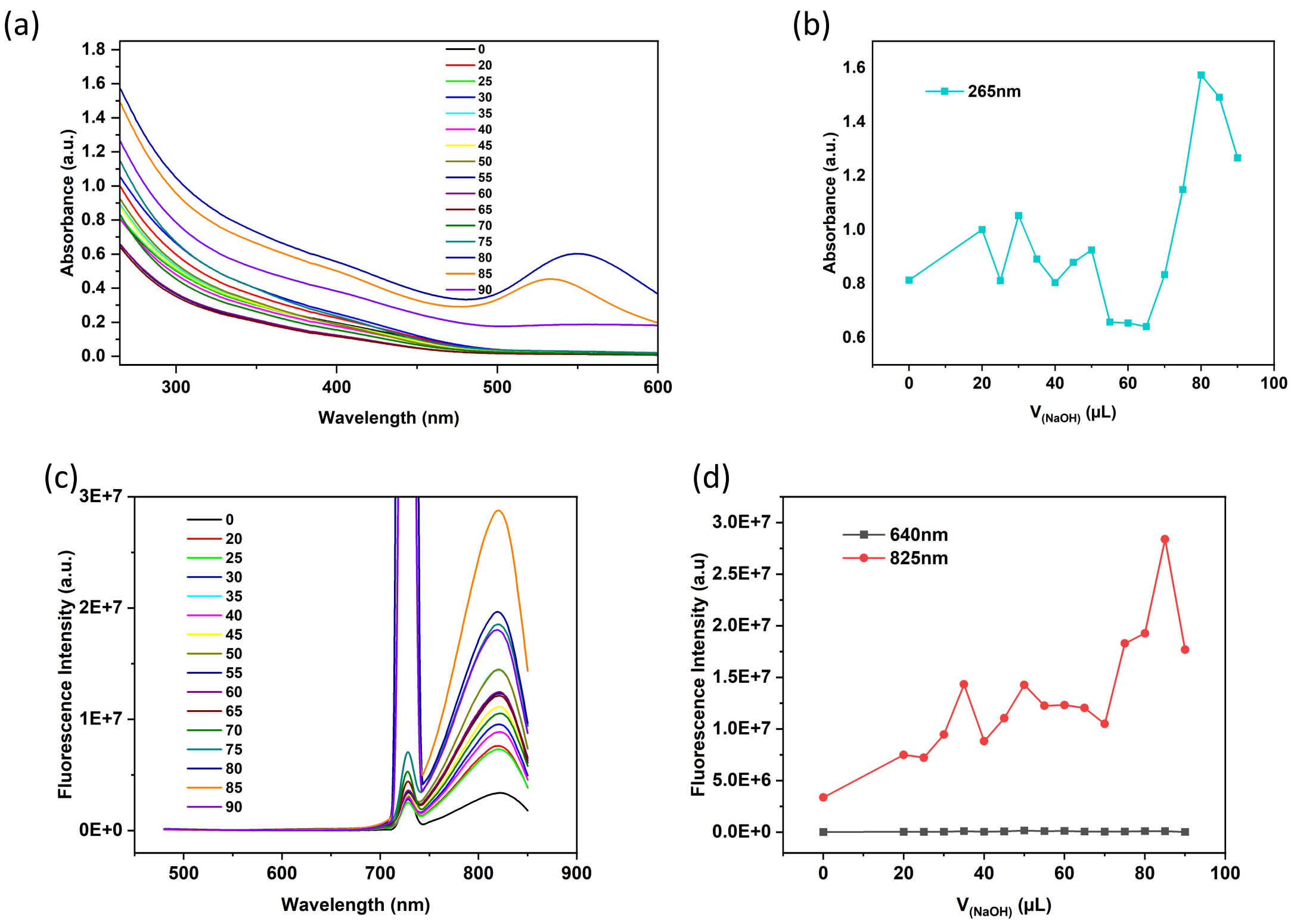

Figure S14. UV-Vis absorption spectra(a), absorbance comparison at 265nm(b), emission spectra(c) and intensity comparison at 640nm and 825nm(d) for GSH-AuNCs synthesised at 95°C, $C_{(Au)}$=34mM, different pH.

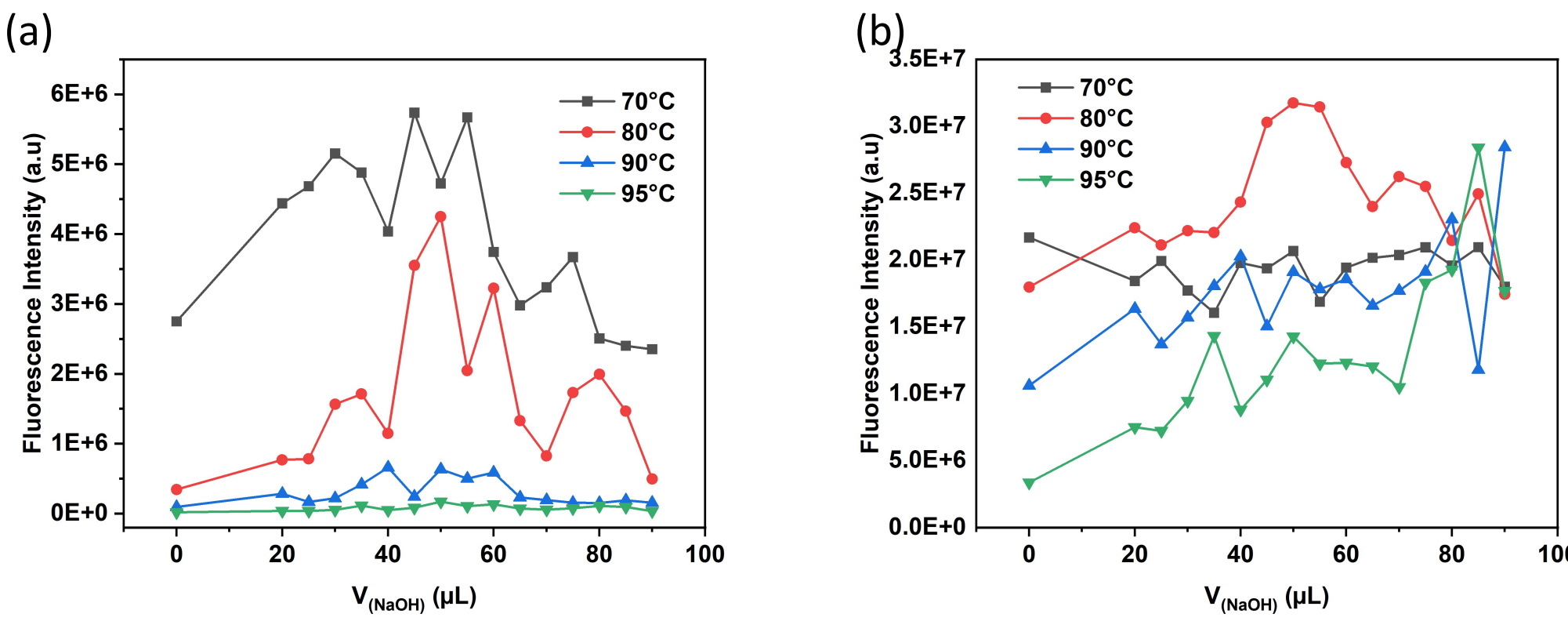

Figure S15. fluorescence intensity comparison of GSH-AuNCs synthesised at different temperatures and different pH with $C_{(Au)}$ = 34mM, (a) emission at 640nm; (b) emission at 825nm

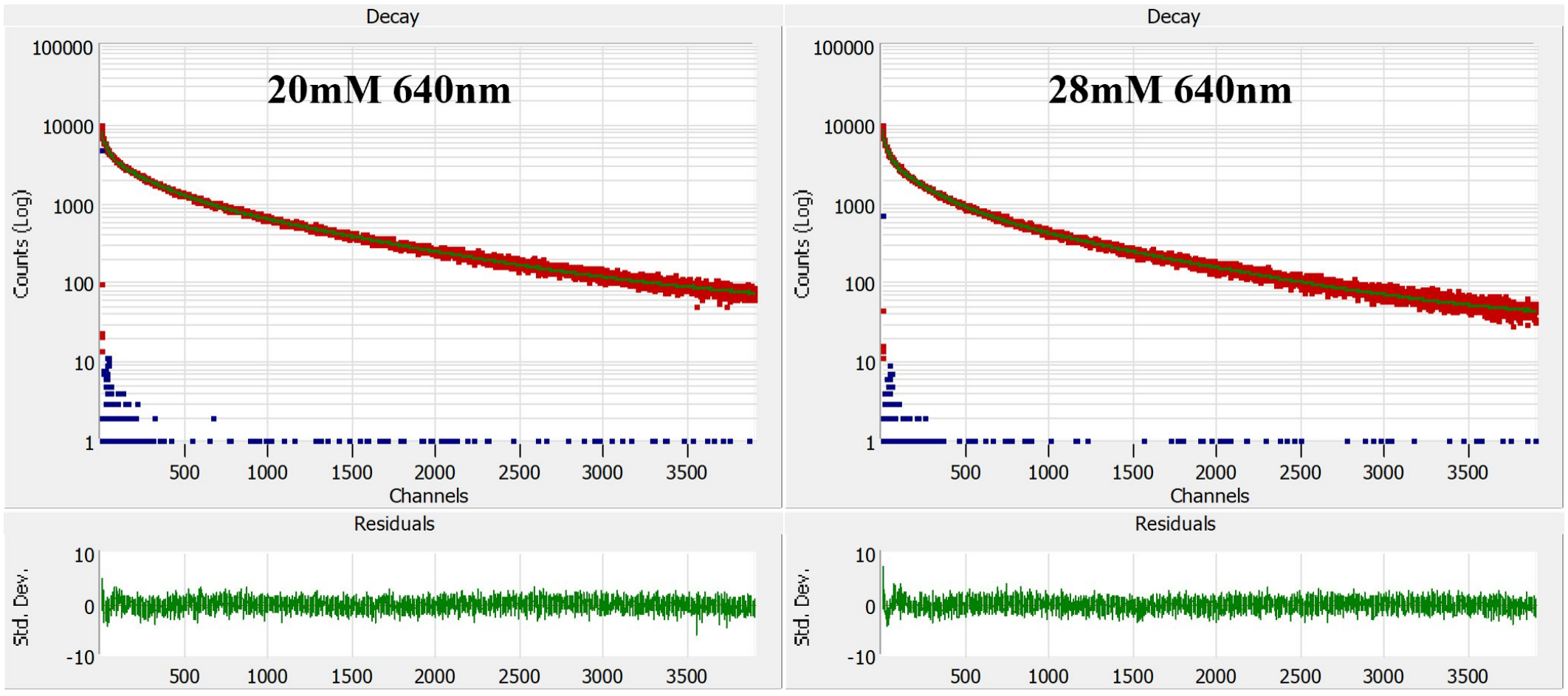

Figure S16. Lifetime decay for (a)red(20mM)/(b)dual(28mM)-emissive GSH-AuNCs at 640nm emission

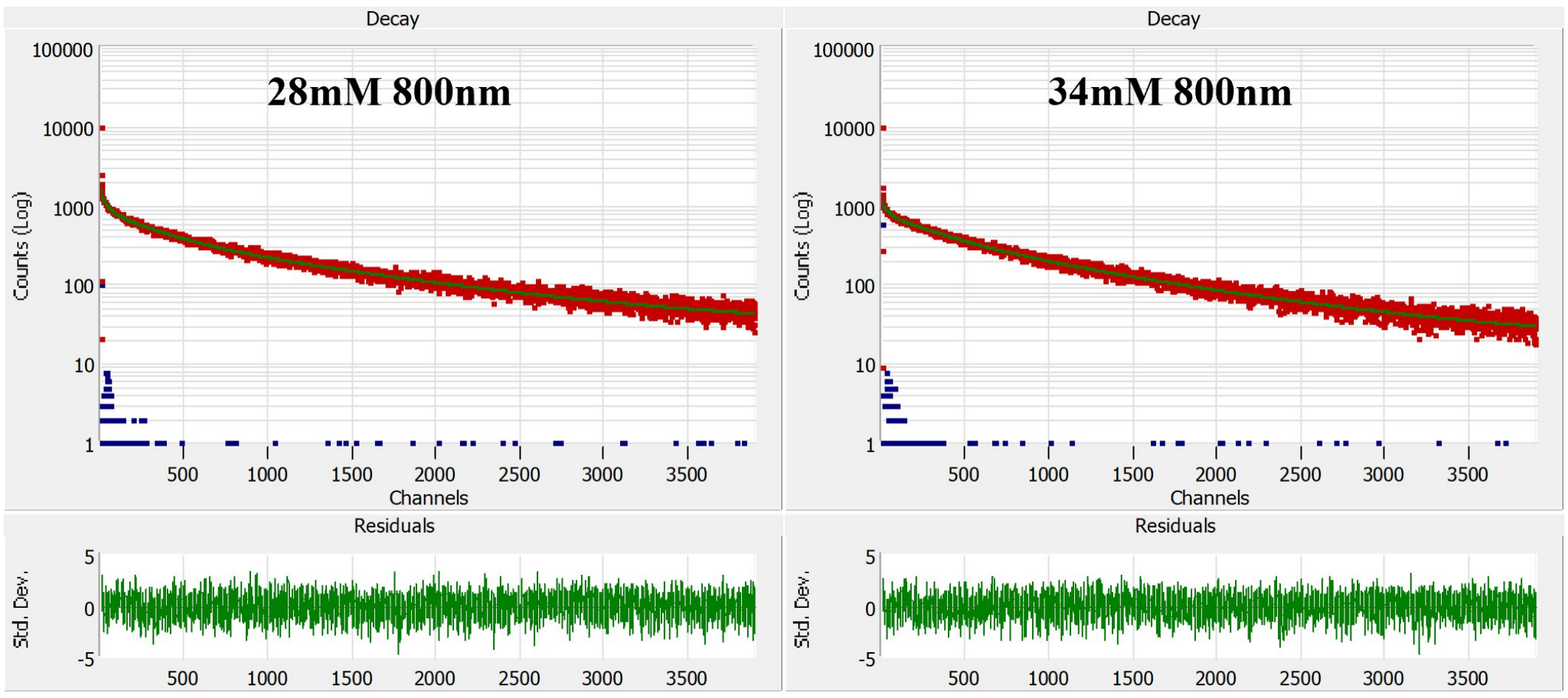

Figure S17. Lifetime decay for (a)dual(28mM)/(b)NIR(34mM)-emissive GSH-AuNCs at 800nm emission

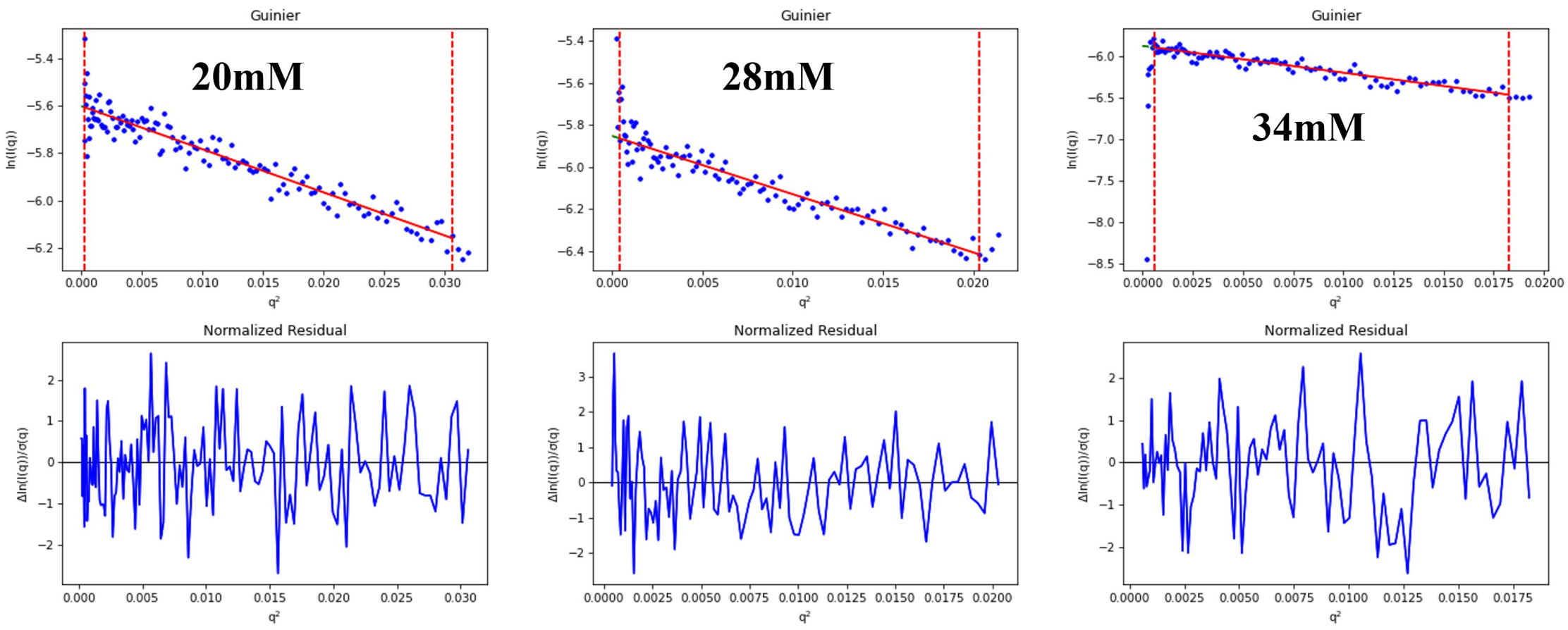

Figure S18. SAXS fitted decay for (a-20mM) red/(28mM) dual/(c-34mM) NIR-emissive GSH-AuNCs
